# Involvement of the IL-6 Signaling Pathway in the Anti-Anhedonic Effect of the Antidepressant Agomelatine in the Chronic Mild Stress Model of Depression

**DOI:** 10.3390/ijms232012453

**Published:** 2022-10-18

**Authors:** Andrea C. Rossetti, Maria Serena Paladini, Cesar Augusto Brüning, Vittoria Spero, Maria Grazia Cattaneo, Giorgio Racagni, Mariusz Papp, Marco A. Riva, Raffaella Molteni

**Affiliations:** 1 Department of Medical Biotechnology and Translational Medicine, University of Milan, 20129 Milan, Italy; 2 Center for Chemical, Pharmaceutical and Food Sciences (CCQFA), Federal University of Pelotas, Pelotas 96010-900, RS, Brazil; 3 Department of Pharmacological and Biomolecular Sciences, University of Milan, 20133 Milan, Italy; 4 Maj Institute of Pharmacology, Polish Academy of Sciences, 31-343 Krakow, Poland; 5 Biological Psychiatry Unit, IRCCS Istituto Centro San Giovanni di Dio Fatebenefratelli, 25125 Brescia, Italy

**Keywords:** stress, major depressive disorder, prefrontal cortex, neuroinflammation, SOCS3

## Abstract

Neuroinflammation has emerged as an important factor in the molecular underpinnings of major depressive disorder (MDD) pathophysiology and in the mechanism of action of antidepressants. Among the inflammatory mediators dysregulated in depressed patients, interleukin (IL)-6 has recently been proposed to play a crucial role. IL-6 activates a signaling pathway comprising the JAK/STAT proteins and characterized by a specific negative feedback loop exerted by the cytoplasmic protein suppressor of cytokine signalling-3 (SOCS3). On these bases, here, we explored the potential involvement of IL-6 signaling in the ability of the antidepressant drug agomelatine to normalize the anhedonic-like phenotype induced in the rat by chronic stress exposure. To this aim, adult male Wistar rats were subjected to the chronic mild stress (CMS) paradigm and chronically treated with vehicle or agomelatine. The behavioral evaluation was assessed by the sucrose consumption test, whereas molecular analyses were performed in the prefrontal cortex. We found that CMS was able to stimulate IL-6 production and signaling, including SOCS3 gene and protein expression, but the SOCS3-mediated feedback-loop inhibition failed to suppress the IL-6 cascade in stressed animals. Conversely, agomelatine treatment normalized the stress-induced decrease in sucrose consumption and restored the negative modulation of the IL-6 signaling via SOCS3 expression and activity. Our results provide additional information about the pleiotropic mechanisms that contribute to agomelatine’s therapeutic effects.

## 1. Introduction

Major depressive disorder (MDD) is a highly prevalent psychiatric disorder associated with great personal distress and massive economic burdens on families and societies [1]. Currently, standard therapies fail to reach the complete remission of the pathology in many patients, thus suggesting an urgent need for new therapeutic targets and a better understanding of the molecular basis of MDD [2].

In the last years, a central role for neuroinflammation in the pathogenesis of MDD has been proposed [3,4]. Compelling evidence includes the measurement of increased levels of inflammatory cytokines in depressed patients [5,6,7] and the capability of anti-inflammatory drugs to reduce depressive symptoms [8,9].

Among cytokines, interleukin (IL)-6 is consistently increased in blood samples of MDD patients [5,10,11]. IL-6 is a pleiotropic cytokine that exerts pro- or anti-inflammatory effects [12] through two main downstream pathways, i.e., the Janus kinase/signal transducer and activator of transcription (JAK/STAT) pathway and the JAK-SH2 domain tyrosine phosphatase 2 (SHP2)-mitogen-activated protein kinase (JAK/MAPK) pathway. The IL-6 signaling cascade is started by the binding of the cytokine to the membrane-bound or soluble form of its receptor (IL-6R and sIL-6R, respectively) followed by the recruitment of the signal transducer gp130 and the activation of the gp130-associated JAK. Once activated, JAK phosphorylates STAT3 and induces the formation of STAT3 homodimers that are able to translocate into the nucleus to transcriptionally regulate IL-6 responsive genes, including the expression of two cytokine receptor signaling inhibitors, SOCS1 and SOCS3, which terminate the IL-6 signaling. SOCS1 directly binds to the activated JAK, thereby reducing its catalytic activity, whereas SOCS3 binds to the phosphorylated gp130 at tyrosine 759 and terminates JAK activation through a negative feedback mechanism. Tyrosine 759 motif is also crucial for the JAK/MAPK pathway since it is the docking site for the tyrosine phosphatase SHP2, which initiates the MAPK pathway. The pro- or anti-inflammatory responses are predominantly mediated by trans- or classic signaling via the sIL-6R or the membrane-bound IL-6R, respectively [13]. These opposite effects are not due to different molecular mechanisms but depend on the expression ratios of sIL-6R or IL-6R to gp130 [14,15]. Notably, the serum levels of sIL-6R have been found significantly higher in treatment-resistant MDD patients than in responsive patients [16].

Agomelatine is an atypical antidepressant with agonist properties at melatonin MT1 and MT2 receptors and antagonist properties at 5-hydroxytryptamine 2C (5-HT2C) receptors [17]. These properties act in a complementary and possibly synergistic manner to reduce depressive symptoms by restoring circadian rhythms and enhancing dopamine and noradrenaline release at the frontal cortex, as well as through other still unknown mechanisms. Remarkably, an immunomodulatory effect of agomelatine on pro-inflammatory cytokines, including IL-6, has been demonstrated by our group in rats treated with lipopolysaccharide [18,19] or exposed to chronic mild stress (CMS) [20], a well-established model of depression [21].

In this study, we investigated whether a modulation of the IL-6 signaling cascade may be involved in the effect of agomelatine in the rat model of CMS. To this aim, we analyzed the gene and protein expression of IL-6 and the activation of the main downstream molecules involved in the cytokine-induced pathway, i.e., JAK1, STAT3, and SOCS3, in brains from CMS-exposed animals treated or not with the antidepressant agomelatine. Among the brain regions, we focused our attention on the prefrontal cortex since chronic stress-induced molecular and structural changes in this area have been involved in the etiopathology of MDD [22].

## 2. Results

### 2.1. Evaluation of Agomelatine Effect on the Impaired Sucrose Intake of CMS Rats

**Figure 1:**
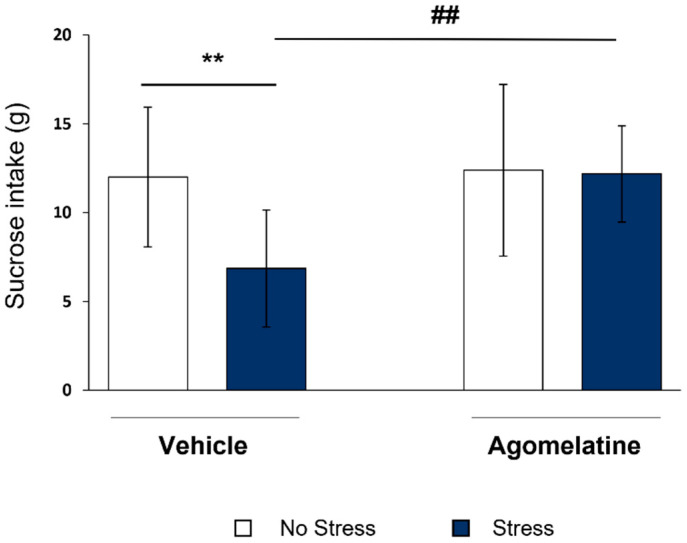
Evaluation of agomelatine effect on impaired sucrose intake of CMS rats. Sucrose intake was assessed after chronic treatment with agomelatine (dark blue bars) in control rats (No Stress/Vehicle) or exposed to chronic mild stress (CMS) (*n* = 10 for each experimental group). Data are expressed in grams (g) of sucrose solution taken by each individual animal in every experimental group. ** *p* < 0.01 No Stress/Vehicle; ## *p* < 0.01 vs. Stress/Vehicle (two-way ANOVA with PLSD).

In line with our previous studies, the exposure to seven weeks of CMS led to the development of an anhedonic-like phenotype, with a significant decrease in sucrose consumption in stressed rats with respect to the control group (F_1,40_ = 5.015, *p* < 0.05; −5.1 g of sucrose solution, *p* < 0.01 vs. No Stress/Vehicle; Figure 1). Chronic treatment with agomelatine was able to normalize the stress-induced alteration (F_1,40_ = 5.753, *p* < 0.05; +5.3 g vs. Stress/Vehicle, *p* < 0.01; Figure 1), an effect sustained by the significant Stress × Agomelatine statistical interaction (F_1,40_ = 4.259, *p* < 0.05). A detailed time course of the effect of the drug on sucrose consumption in rats exposed to CMS is shown in Figure S1. Considering the timing of the molecular analyses, here we showed the behavioral readout of our experimental paradigm only at the last time point (week 7).

### 2.2. Modulation of IL-6 mRNA and Protein Levels and JAK1 Activation in Rats Exposed to CMS and Treated with Agomelatine

**Figure 2:**
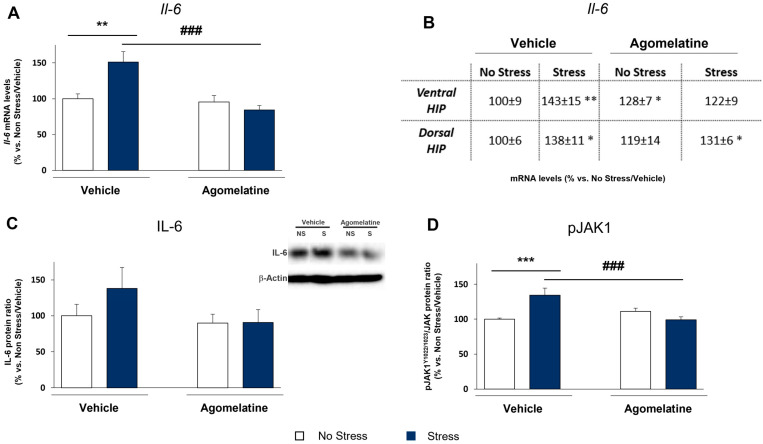
Modulation of IL-6 mRNA and protein levels and JAK1 activation in rats exposed to CMS and treated with agomelatine. mRNA levels of IL-6 were assessed in prefrontal cortex (**A**) and in ventral and dorsal subregions of hippocampus (**B**). Protein levels of IL-6 and activation of JAK1 (ratio at tyrosine 1022/1023) were measured in prefrontal cortex of rats exposed to CMS and/or to chronic treatment with agomelatine (**C**,**D**). Data, expressed as percentage vs. unstressed rats treated with vehicle (No Stress/Vehicle, set at 100%), are mean ± SEM of at least 7 independent determinations. * *p* < 0.05, ** *p* < 0.01, *** *p* < 0.001 vs. No Stress/Vehicle; ### *p* < 0.001 vs. Stress/Vehicle (two-way ANOVA with PLSD).

The ability of agomelatine to counteract the CMS-induced anhedonic phenotype prompted us to investigate the underlying molecular mechanism(s). In view of the proposed immunomodulatory effect of the drug, we first studied whether the treatment with agomelatine might regulate the IL-6 expression and signaling pathway in the CMS model. As shown in Figure 2A, IL-6 mRNA levels were upregulated in the prefrontal cortex of stressed rats (+51% vs. No Stress/Vehicle, *p* < 0.01), an effect that was normalized by agomelatine treatment (67% vs. Stress/Vehicle, *p* < 0.001) as indicated by a significant Stress × Agomelatine interaction (F_1,32_ = 9.868, *p* < 0.01). Conversely, the stress-induced increase in IL-6 in both ventral and dorsal hippocampal regions (+43% and +38%, respectively, vs. No Stress/Vehicle, *p* < 0.01; Figure 2B,C) was not affected by the pharmacological treatment. Based on this anatomical specificity, we focused our further analyses on the prefrontal cortex by measuring IL-6 protein levels. Similar to what observed at transcriptional level, CMS upregulated IL-6 protein (+38% vs. No Stress/Vehicle; Figure 2D), although the increase did not reach statistical significance. However, when the stressed animals were treated with agomelatine, the rise in IL-6 levels was totally blunted (−47% vs. Stress/Vehicle, *p* > 0.05; Figure 2D).

We next evaluated the activation of JAK1, an accessory protein that mediates the cascade resulting from the interaction between IL-6 and its membrane receptor. The results of the analysis of the active form of JAK1 phosphorylated at tyrosine 1022 and 1023 showed a stress-dependent JAK1 stimulation (+34% vs. No Stress/Vehicle, *p* < 0.001; Figure 2) that was normalized by the pharmacological treatment (−35% vs. Stress/Vehicle, *p* < 0.001; Figure 2), with a significant Stress × Agomelatine interaction (F_1,32_ = 15.135, *p* < 0.001). Collectively, our data confirm the increase in IL-6 levels and the activation of its downstream pathway in the prefrontal cortex of rats exposed to CMS. Of note, agomelatine appeared to be able to modulate either the CMS-induced increase in IL-6 or the intracellular pathway stimulated by the cytokine.

### 2.3. Modulation of STAT3 Activation in the Prefrontal Cortex of Rats Exposed to CMS and Treated with Agomelatine

**Figure 3:**
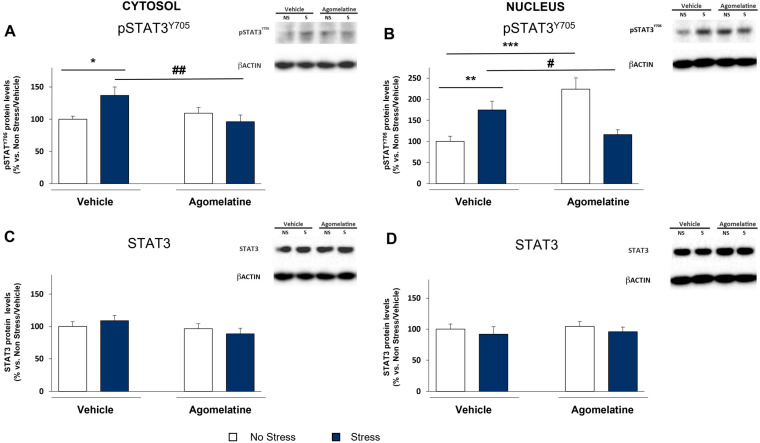
Modulation of STAT3 activation in prefrontal cortex of rats exposed to CMS and agomelatine treatment. Protein levels of phosphorylated (Tyr705) and total forms of STAT3 were assessed in cytosol (**A**,**C**) and in nucleus (**B**,**D**). All analyses were conducted in prefrontal cortex of rats exposed to CMS and chronic treatment with agomelatine. Data, expressed as percentage vs. unstressed rats treated with vehicle (No Stress/Vehicle, set at 100%), are mean ± SEM of at least 7 independent determinations. * *p* < 0.05; ** *p* < 0.01; *** *p* < 0.001 vs. No Stress/Vehicle; # *p* < 0.05, ## *p* < 0.01 vs. Stress/Vehicle (two-way ANOVA with PLSD).

To better evaluate the impact of stress and agomelatine on IL-6 signaling, we analyzed the activation of key proteins recruited in this pathway, starting from STAT3—the downstream target of JAK1—by measuring its phosphorylated form at tyrosine 705 (pSTAT3^Y705^) that represents the active form of the protein [23]. The analyses were run in the cytosol as well as in the nucleus, where pSTAT3^Y705^ acts as a transcription factor. As shown in Figure 3A, the stress-induced upregulation of IL-6 was paralleled by the increased levels of pSTAT3^Y705^ in the cytosol (+37% vs. No Stress/Vehicle, *p* < 0.05; Figure 3A). Interestingly, agomelatine was able to normalize this effect (−41% vs. Stress/Vehicle, *p* < 0.01; Figure 3A) without any modulation of pSTAT3^Y705^ in unstressed animals, as indicated by a significant Stress × Agomelatine interaction (F_1,36_ = 6.764, *p* < 0.05). No changes in the expression of the total form of STAT3 were observed in any experimental groups (Figure 3C).

In line with the increased phosphorylation of STAT3 in the cytosolic compartment, the levels of pSTAT3^T705^ were upregulated by stress also in the nucleus (+75% vs. No Stress/Vehicle, *p* < 0.01; Figure 3B). Interestingly, agomelatine treatment showed a different impact on unstressed vs. stressed animals, as supported by the significant Stress × Agomelatine interaction (F_1,27_ = 23.212, *p* < 0.001). Indeed, while the antidepressant strongly upregulated pSTAT3^Y705^ levels in unstressed rats (+124% vs. No Stress/Vehicle, *p* < 0.001; Figure 3B), it hampered the activation of the transcription factor in stressed rats (−59% vs. Stress/Vehicle, *p* < 0.05; Figure 3B). Again, no changes were found in the total form of STAT3 (Figure 3D).

Therefore, the phosphorylation of STAT3 observed in CMS-exposed rats validates the presence of an active IL-6 pathway in the prefrontal cortex of stressed animals. The ability of agomelatine to decrease STAT3 phosphorylation favors its involvement in the negative modulation of the CMS-induced IL-6 signaling. At variance, agomelatine itself phosphorylated STAT3 in unstressed animals, suggesting the capacity of the drug to activate STAT3 via other mechanisms, independent from the IL-6/JAK1 axis.

### 2.4. Modulation of SOCS3 Expression in the Prefrontal Cortex of Rats Exposed to CMS and Treated with Agomelatine

**Figure 4:**
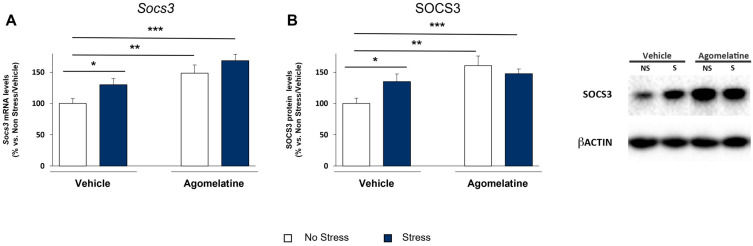
Modulation of SOCS3 levels in prefrontal cortex of rats exposed to CMS and treated with agomelatine. mRNA (**A**) and protein (**B**) levels of SOCS3 were assessed in prefrontal cortex of rats exposed to CMS and treatment with agomelatine. Data, expressed as percentage vs. unstressed rats treated with vehicle (No Stress/Vehicle, set at 100%), are mean ± SEM of at least 8 independent determinations. * *p* < 0.05, ** *p* < 0.01, *** *p* < 0.001 vs. No Stress/Vehicle (two-way ANOVA with PLSD).

We then evaluated whether the nuclear modulation of pSTAT3^Y705^ was paralleled by functional changes in its activity by measuring the mRNA levels of SOCS3, a transcriptional target of STAT3 involved in IL-6 signaling negative feedback loop. In accordance with STAT3 activation, *Socs3* gene expression was enhanced by both stress (F_1,33_ = 6.216, *p* < 0.05) and agomelatine (F_1,33_ = 11.077, *p* < 0.01). Specifically, as depicted in Figure 4A, *Socs3* mRNA levels were significantly increased in CMS rats (+30% vs. No Stress/Vehicle, *p* < 0.05), and a greater effect was observed after agomelatine administration in unstressed animals (+49% vs. No Stress/Vehicle, *p* < 0.01). The results of the analysis of SOCS3 protein in the cytosolic compartment (Figure 4B) showed a similar profile, with an overall increase by stress exposure (+35% vs. No Stress/Vehicle, *p* < 0.05) and by agomelatine in unstressed animals (+61% vs. No Stress/Vehicle, *p* < 0.001). Remarkably, a significant increase in both *Socs3* gene and protein was observed in agomelatine-treated stressed rats (+48% vs. No Stress/Vehicle, *p* < 0.01, and +69% vs. No Stress/Vehicle, *p* < 0.001, per mRNA and protein levels, respectively) in the absence of any upstream STAT3 activation. At variance with the prefrontal cortex, we did not observe any increase in the *Socs3* gene expression in the dorsal and ventral hippocampus.

### 2.5. MAP Kinases as Alternative Mechanisms of STAT3/SOCS3 Activation in Prefrontal Cortex of Rats Exposed to CMS and Treated with Agomelatine

According to our analyses, the increased SOCS3 expression observed in stressed animals treated with agomelatine was not accompanied by an upstream activation of the IL-6 pathway, including STAT3 phosphorylation at tyrosine 705. Therefore, in the attempt to understand whether other mechanisms could be responsible for this SOCS3 upregulation, we investigated other pathways that are able to modulate STAT3 transcriptional activity and/or induce SOCS3 expression. We found that neither stress nor agomelatine had an impact on nuclear pSTAT3^S727^ levels (Figures S2–S17), thus excluding the involvement of this alternative mechanism of STAT3 transcriptional activation in the downstream increase in SOCS3.

**Figure 5:**
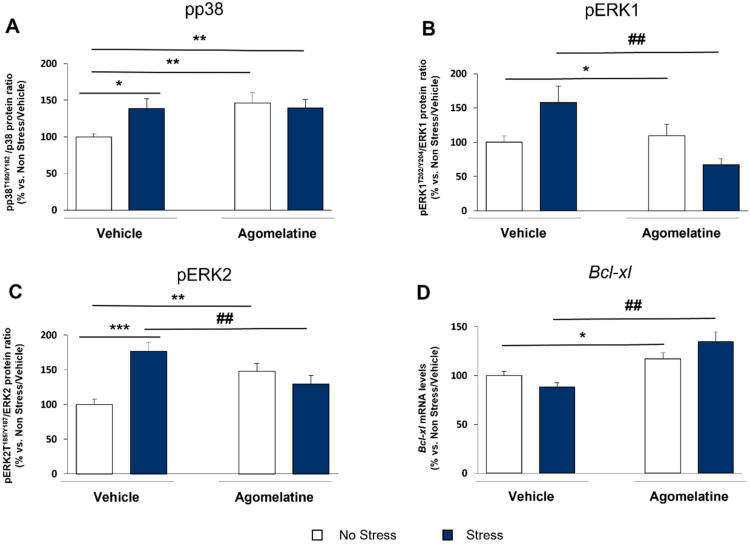
MAP kinases as alternative mechanisms of STAT3/SOCS3 activation in prefrontal cortex of rats exposed to CMS and treated with agomelatine. Activation ratio of nuclear p38 at Threonine180/Tyrosine182 (**A**) and ERK1/2 at Threonine202/Tyrosine204 (**B**,**C**), as well as mRNA levels of the *BCL-xl* gene (**D**) were assessed in prefrontal cortex of rats exposed to CMS and treated with agomelatine. Data, expressed as percentage vs. unstressed rats treated with vehicle (No Stress/Vehicle, set at 100%), are mean ± SEM of at least 6 independent determinations and are presented as a ratio between phosphorylated and total forms of proteins examined. * *p* < 0.05; ** *p* < 0.01; *** *p* < 0.001 vs. No Stress/Vehicle; ## *p* < 0.01 vs. Stress/Vehicle (two-way ANOVA with PLSD).

It is well-known that STAT3 can also be activated via phosphorylation at serine 727 by members of the mitogen-activated protein kinases (MAPKs), such as p38 MAPK (p38) and extracellular signal-regulated kinases (ERK1 and ERK2). Therefore, we assessed the activation of these proteins by measuring their phosphorylated forms. As shown in Figure 5, CMS significantly induced the activation of p38 (+46% vs. No Stress/Vehicle, *p <* 0.01; Figure 5A), ERK1 (+58% vs. No Stress/Vehicle, *p* < 0.05; Figure 5B), and ERK2 (+77% vs. No Stress/Vehicle, *p* < 0.001; Figure 5C). Notably, the activation of p38 was significantly upregulated by CMS also in rats treated with agomelatine (+39% vs. No Stress/Vehicle, *p* < 0.05) in accordance with the results previously described for SOCS3 expression. Conversely, the antidepressant was able to normalize the stress-induced activation of ERK1 and ERK2 to control levels (−91% and −47% vs. Stress/Vehicle, *p* < 0.01), as indicated by a significant Stress × Agomelatine interaction (F_1,30_ = 8.406, *p* < 0.001, and F_1,30_ = 17.778, *p* < 0.001, respectively). Remarkably, agomelatine per se differentially modulated the activity of the kinases: ERK1 was unaffected (Figure 5B), whereas a significant increase was observed for both p38 (+46%, *p* < 0.01 vs. No Stress/Vehicle) and ERK2 (+48%, *p* < 0.01 vs. No Stress/Vehicle). These data support the idea that different molecular mechanisms might sustain the activity of the drug in a physiological or stressful behavioral context.

Searching for possible unknown targets of the agomelatine-mediated MAPK modulation, we evaluated the mRNA levels of the antiapoptotic gene *Bcl*-*xl.* Here, we found that the expression of *Bcl-xl* was not altered by CMS exposure but significantly upregulated by agomelatine (F_1,37_ = 16.929, *p* < 0.001; Figure 5D) in unstressed (+17% vs. No Stress/Vehicle, *p* < 0.05) as well as stressed rats (+45% vs. Stress/Vehicle, *p* < 0.001), thus suggesting that an antiapoptotic and neuroprotective function might be part of the pharmacological activity of the drug.

## 3. Discussion

Our study provided evidence that the capability of the antidepressant agomelatine to revert the anhedonic-like phenotype observed in the CMS model of depression is associated to the modulation of IL-6 levels and signaling. Specifically, we show that (1) chronic stress upregulates IL-6 and activates the associated JAK/STAT3 pathway leading to increased levels of SOCS3 protein, with the latter effect, however, unable to inhibit IL-6 signaling by negative feedback; (2) at variance, the agomelatine-induced increase in SOCS3 is able to dampen the activation of IL-6 pathway in stressed animals; and (3) stress and agomelatine are both capable to increase SOCS3 expression but through different and still unknown molecular mechanisms.

In agreement with our previous work [20], here, we confirmed that chronic stress increases the expression of IL-6 in the rat prefrontal cortex, a brain area particularly vulnerable to stressful conditions [24]. This finding is supported by preclinical studies showing elevated levels of IL-6 in response to different stress paradigms, in the brain [25,26] as well as in the periphery [27,28]. The involvement of stress-induced IL-6 increase in depression-like behaviors has been demonstrated by using IL-6 knockout mice [29], and a strong association between IL-6 alterations and major depressive disorder has also been shown at clinical level [5,30]. In this context, the ability of agomelatine to counteract the increase in IL-6 may have an important translational impact. Indeed, we have previously demonstrated that agomelatine has anti-inflammatory properties in the brain [18,19,20], and analogous studies have been performed with other antidepressants [31,32,33]. In the present study, we provided new information on the mechanism of action of agomelatine in the context of stress-induced neuroinflammation. As previously observed [17], we found that the drug was able to modulate inflammatory mediators not only in stressful conditions but also in a control situation, an effect in line with the inherent protective and anti-inflammatory properties of the antidepressant. As a step forward, here we showed that this occurs through two different putative molecular mechanisms. Indeed, the similar modulation of the feedback inhibitor of IL-6 cascade, SOCS3, observed in both basal and stressed conditions, was respectively associated to changes of pSTAT3 and MAPK.

Importantly, we demonstrated that chronic stress stimulates not only IL-6 production but also its downstream pathway, starting from the activation of the receptor-bound protein JAK1, then through the cytoplasmic and nuclear activation of STAT3, and finally triggering the gene and protein expression of SOCS3. This protein physiologically acts as a feedback-loop inhibitor either targeting the phosphorylation site of JAK1 or blocking the cytosolic activation of STAT3 [34,35]. Surprisingly, in untreated stressed animals, SOCS3 did counteract neither the phosphorylation of JAK1 nor the activation of STAT3 via phosphorylation at the Tyr705 residue, despite its expression being increased by stress. The dysregulation of the SOCS3-dependent inhibition loop might be due to the long-lasting overactivation of the IL-6 signaling pathway that accompanied stressful conditions—that is, in our study, the exposure to CMS—leading to the establishment of a chronic detrimental setting and to the lack of physiological blockade and adaptive responses. To favor this hypothesis, a functional alteration of IL-6 signaling, with an increased expression of IL-6 receptor and SOCS3, has been shown in fibroblasts from depressed subjects [36]. In addition, an abnormal expression and function of SOCS has been observed in cytokine-producing immune cells in the tumor microenvironment, another biological niche where a persistent production of inflammatory cytokines might disrupt physiological negative feedback mechanisms [37]. At variance, in the presence of agomelatine, the SOCS3-mediated feedback is still present in stressed animals. Therefore, the ability of agomelatine to counteract the activation of the IL-6-induced JAK/STAT pathway by maintaining the feedback activity of SOCS3 might actually be due to a reduction of the cytokine levels induced by the drug treatment.

Very interestingly, the ability of agomelatine to induce SOCS3 expression might represent a mechanism shared with other psychotropic drugs in the regulation of the JAK/STAT pathway. In this regard, Al-Samhari and collaborators showed that an acute treatment with fluoxetine after forced swim stress was able to increase *Socs3* mRNA levels [38], and a similar effect was found in the hippocampus of mice exposed to chronic stress and chronically treated with the selective serotonin reuptake inhibitor (SSRI) [39]. Unfortunately, only few studies have been focused on the pharmacological modulation of SOCS3 at the level of the CNS, and the associated discordant results are probably due to differences in the experimental conditions, including stress paradigms, brain regions, and time points for analyses [40]. Nevertheless, the fact that drugs belonging to different families (e.g., agomelatine, SSRI) may converge on the modulation of SOCS3 suggests that its expression may be transcriptionally regulated through multiple independent mechanisms in addition to the canonical STAT3 activation. As an example, the increased expression of SOCS3 in CMS animals could contribute to the activity of MAP kinases such as ERK1/2 and p38, which have been involved in the *Socs3* transcription in the absence of STAT3 [41]. These enzymes are able to translocate into the nucleus and activate transcription factors whose consensus regions may lie on the promoter of *Socs3*, as demonstrated in human non-neuronal cell lines [42]. Accordingly, chronic stress activated nuclear p38, ERK1, and ERK2 in our CMS-exposed animals, thus suggesting additional mechanisms that might contribute to the SOCS3 expression and a compromised feedback inhibitory regulation. Further analyses are, however, necessary to more deeply understand the role of MAP kinases in the differential modulation of IL-6 signaling, since the control of SOCS3 expression by these enzymes has been only partially resolved [43].

There is an unsolved question regarding how agomelatine can induce a robust and functional SOCS3 expression without a concurrent STAT3 phosphorylation on Tyr705, especially in the cytosol. However, as described above, other mechanisms have been suggested to regulate SOCS3 expression. To this regard, agomelatine is a well-known agonist at the MT1 and MT2 receptors [17], and these receptors activate multiple signaling pathways including MAPK [44]. Although further studies are demanded, we can suggest that the agonistic activity of agomelatine on MT1/MT2 receptors might contribute to the SOCS3 increase and, finally, to the pharmacological action of the drug.

Importantly, we observed that the treatment with agomelatine not only reverted the behavioral alterations in stressed rats but also increased the mRNA levels of *Bcl-xl.* This antiapoptotic gene was induced only by the drug, both in the basal condition as well as after stress exposure. Bcl-xL is known to have a pronounced neurotrophic effect supporting neuronal survival [45], and its modulation by the pharmacological treatment is in agreement with the reported antiapoptotic properties of agomelatine itself [46,47] and of other antidepressants [48,49,50]. Thus, in addition to the anti-inflammatory properties, agomelatine also possesses a neuroprotective role that might act together to normalize the stress-induced activation of the IL-6 pathway and its deleterious effects. In the context of the antiapoptotic effect of the drug, and in support of the possibility of it being carried out through MT1/MT2 receptors and the activation of MAPKs, we observed a selective activation of p38 in animals treated with agomelatine. Interestingly, although in different frameworks, p38 has been demonstrated to induce at the transcriptional level both *Bcl2* and *Bcl-xl* antiapoptotic genes [51,52].

Lastly, it should be mentioned that the data presented in this study suffered from a few limitations. Indeed, we designed our study with a single 24 h analytic time point following the last session of CMS, which yields to a partial picture of the whole responsiveness to stress and to the pharmacological treatment. Indeed, different mediators might have a specific time-dependent modulatory profile. Moreover, changes at the translational level might occur later with respect to those at the transcriptional level. On these bases, it will be worthwhile to further investigate the effects of agomelatine on IL-6 expression and signaling with an experimental design that includes multiple time points.

In conclusion, our work highlighted the complexity of agomelatine effect in controlling the stress-induced IL-6 upregulation and pathway in the CMS model that can act in concert with the modulatory effect of the drug on other pro-inflammatory cytokines [18,20]. Multiple molecular mechanisms are involved in the anti-inflammatory activity of the drug within the brain, as well as in its capability to ameliorate depressive-like behaviors. In addition to the anti-inflammatory properties, we also proposed a protective mechanism for agomelatine mediated by its ability to induce the antiapoptotic gene *Bcl-xl*. Despite the fact that future studies will be necessary to shed light on the mechanism of action of agomelatine, our data strongly support the requirement to further investigate the impact of antidepressant drugs on cytokine signaling in order to better understand how psychotropic drugs may impact neuroinflammation as a promising therapeutic target for psychiatric disorders.

## 4. Material and Methods

### 4.1. Animals

Two-month-old male Wistar rats (Charles River, Sulzfeld, Germany) were brought into the laboratory one month before the start of the experiment. Except as described below, the animals were singly housed with food and water freely available and maintained on a 12 h light–dark cycle in constant temperature (22 ± 2 °C) and humidity (50 ± 5%) conditions. All procedures used in this study conformed to the rules and principles of the 2010/63/EU Directive and were approved by the Local Bioethical Committee at the Institute of Pharmacology, Polish Academy of Sciences, Krakow, Poland (authorization number 1272/2015). All efforts were made to minimize animal suffering and reduce the number of animals used.

### 4.2. Stress Procedure and Pharmacological Treatment

After a period of adaptation to laboratory and housing conditions, the animals were trained to consume a 1% sucrose solution. Training consisted of nine 1 h baseline tests in which sucrose was presented, in the home cage, following 14 h of food and water deprivation. The sucrose intake was measured at the end of the test by weighing preweighed bottles containing the sucrose solution. Subsequently, sucrose consumption was monitored under similar conditions at weekly intervals throughout the whole experiment. On the basis of their sucrose intake in the final baseline test, the animals were divided into two matched groups to be subjected to a chronic mild stress procedure [53] for a period of seven weeks. Each week of the stress regime consisted of two periods of food or water deprivation, two periods of 45 degrees cage tilt, two periods of intermittent illumination (lights on and off every 2 h), two periods of soiled cage (250 mL water in sawdust bedding), one period of paired housing, two periods of low-intensity stroboscopic illumination (150 flashes/min), and three periods of no stress. All stressors were 10–14 h of duration and were individually and continuously applied, day and night. Control animals were housed in separate rooms and had no contact with the stressed animals. They were deprived of food and water for 14 h preceding each sucrose test, but, otherwise, food and water were freely available in the home cage.

Based on the results of the final sucrose test carried out following the first 2 weeks of stress, both control and stress groups were further divided into matched subgroups. Specifically, only stressed animals with at least 50% of sucrose consumption with respect to their baseline were kept in our experimental design as responsive to the stress procedure. Then the animals received for the subsequent five weeks intraperitoneal injections (i.p.) of vehicle (hydroxyethyl-cellulose; HEC 1%) or agomelatine (40 mg/kg daily). The dose of agomelatine was based on previous studies of our laboratory, showing an efficacy of the treatment on anhedonic-like phenotype [20] and on LPS induced neuroinflammation [18,19]. Both agomelatine and HEC were kindly provided by Servier (Suresnes, France). The stress paradigm was continued throughout the entire period of drug administration. With this experimental design, we obtained four groups: rats that were left undisturbed and received HEC used as the control group (CTRL, *n* = 10); rats exposed to the CMS procedure that received HEC for five weeks (STRESS; *n* = 10); unstressed rats that were chronically administered with agomelatine (AGO; *n* = 10); and rats that were subjected to CMS while being treated with the antidepressant (STRSS/AGO; *n* = 10). After five weeks, the treatments were terminated, and all the animals were sacrificed by decapitation 24 h after the last drug administration, with their brains removed and dissected for ventral and dorsal hippocampus and prefrontal cortex. All samples were then rapidly frozen in dry ice and isopentane and stored at −80 °C. RNA and total proteins were obtained respectively from the two hemispheres of the same brain for each animal.

### 4.3. RNA Preparation and Gene Expression Analyses

For gene expression analyses, the total RNA was isolated from the different brain regions by single-step guanidinium isothiocyanate and phenol extraction using a PureZol RNA isolation reagent (Bio-Rad Laboratories S.r.l.) according to the manufacturer’s instructions and quantified by spectrophotometric analysis. The samples were then processed for real-time polymerase chain reaction (PCR) as previously reported [20] to assess the mRNA levels of *Il-6*, *Socs3*, and *Bcl-xl*.

Briefly, an aliquot of each sample was treated with DNase to avoid DNA contamination and subsequently analyzed by TaqMan qRT-PCR instrument (CFX384 real-time system, Bio-Rad Laboratories S.r.l.) using an iScript one-step RT-PCR kit for probes (Bio-Rad Laboratories S.r.l.). Samples were run in a 384-well format in triplicates as multiplexed reactions with a normalizing internal control (β-Actin and/or GAPDH). Thermal cycling was initiated with incubation at 50 °C for 10 min (RNA retrotranscription) and then at 95 °C for 5 min (TaqMan polymerase activation). After this initial step, 39 cycles of PCR were performed. Each PCR cycle consisted of heating the samples at 95 °C for 10 s to enable the melting process, and then for 30 s at 60 °C for the annealing and extension reactions. A comparative cycle threshold (Ct) method was used to calculate the relative target gene expression. The probe and primer sequences used were purchased from Applied Biosystem Italia and Eurofins MWG-Operon. A complete list of primers and probes used for the gene expression analyses is presented in Table 1.

### 4.4. Protein Extraction, Cellular Fraction Preparation, and Western Blot Analyses

Prefrontal cortices were manually homogenized in a glass–glass potter in an ice-cold 0.32 M sucrose buffer (pH 7.4) containing 1 mM 4-(2-hydroxyethyl)-1-piperazine ethanesulfonic acid (HEPES; Sigma-Aldrich), 0.1 mM ethylene glycol tetra-acetic acid (EGTA; Sigma-Aldrich, St. Louis, MO, USA), and 0.1 mM phenylmethylsulfonyl fluoride in the presence of commercial cocktails of protease (Roche) and phosphatase (Sigma-Aldrich) inhibitors. The homogenate was clarified at 1000× *g* for 10 min. The pellet (P1) was kept as a nuclear fraction and resuspended in a proper buffer (20 mM HEPES, 0.1 mM dithiothreitol, 0.1 mM EGTA) supplemented with protease and phosphatase inhibitors, while the supernatant was centrifuged at 13,000× *g* for 15 min. The resulting supernatant (S2) was recovered as the cytosolic fraction, while the pellet (P2), corresponding to the crude membrane fraction, was resuspended in the resuspension buffer described above. The total protein content was measured according to the Bradford Protein Assay procedure (Bio-Rad Laboratories) using bovine serum albumin as the calibration standard. Equal amounts of protein (15 μg) were run under reducing conditions on SDS-PAGE (polyacrylamide gel electrophoresis) and then electrophoretically transferred onto nitrocellulose or polyvinylidene difluoride (PVDF; Becton Dickinson) membranes. Unspecific binding sites were blocked for 1 h in 10% nonfat dry milk in Tris-buffered saline, and membranes were then incubated overnight with the opportune primary and secondary antibodies in a blocking buffer (the protein analyzed and the conditions of the antibodies used are listed in Table 2). Immunocomplexes were visualized by chemiluminescence using the Western Lightning ECL (Perkin Elmer) and the Chemidoc MP imaging system (BioRad Laboratories). The results were normalized using β-actin as an internal standard (complete immunoblots are provided as Supplementary Material Figures S3–S17).

### 4.5. Statistical Analysis

All the analyses were carried out in individual animals (independent determinations). The effect of the pharmacological treatment was evaluated by two-way ANOVA, with treatment and stress as independent factors. When appropriate, further differences were analyzed by Fisher’s protected least significant difference (PLSD). Significance for all tests was assumed for *p* < 0.05. For graphic clarity, data are presented as means percent ± standard error (SEM) of the control group, i.e., unstressed animals treated with vehicle (No Stress/Vehicle). All the results with the corresponding statistical analyses are listed in Supplementary Table S1.

## Figures and Tables

**Table 1:** Sequences of forward and reverse primers and probes used in qRT-PCR analyses.

*Gene*	Forward Primer	Reverse Primer	Probe
*Socs3*	AGAGCGGATTCTACTGGAGT	TCGACGCTCAGTGTGAAGAA	TTTCTTATCCGCGACAGCTC
*Bcl-xl*	GAACTCTTTCGGGATGGGGTAA	ACTTGCAATCCGACTCACCA	AGCGTAGACAAGGAGATGCA
*β-Actin*	CACTTTCTACAATGAGCTGCG	CTGGATGGCTACGTACATGG	TCTGGGTCATCTTTTCACGGTTGGC
*Il-6*	Purchased from Applied Biosystem (Italy) cod. Rn99999011_m1		
*Gapdh*	Purchased from Applied Biosystem (Italy) cod. Rn99999916_s1		

Abbreviations: qRT-PCR, reverse-transcriptase real-time polymerase chain reaction; *Socs*, suppressor of cytokine signaling; *Bcl-xl*, B-cell lymphoma-extra-large; *Il-6*, inteleukin-6; *Gapdh*, glyceraldehyde 3-phosphate dehydrogenase.

**Table 2:** List of antibodies used in Western blot analyses and incubation conditions.

Target Protein	Primary Antibody	Secondary Antibody
IL-6 (21 kDa)	1:500, BSA 5% in TBS-t Santa Cruz Biotech ID: AB_2127595	HRP conjugated anti-rabbit IgG 1:500 Cell Signaling
pSTAT3 Tyr705 (86 kDa)	1:500, BSA 5% in TBS-t Cell Signaling ID: AB_331586	HRP conjugated anti-rabbit IgG 1:1000 Cell Signaling
pSTAT3 Ser727 (86 kDa)	1:500, BSA 5% in TBS-t Cell Signaling ID: AB_331589	HRP conjugated anti-rabbit IgG 1:1000 Cell Signaling
STAT3 (86 kDa)	1:2000, BSA 5% in TBS-t Cell Signaling ID: AB_331269	HRP conjugated anti-rabbit IgG 1:4000 Cell Signaling
SOCS3 (23 kDa)	1:1000, BSA 5% in TBS-t Cell Signaling ID: AB_2286460	HRP conjugated anti-rabbit IgG 1:1000 Cell Signaling
pJAK1 Tyr1022/1023 (120 kDa)	1:500, BSA 5% in TBS-t Cell Signaling ID: AB_2265057	HRP conjugated anti-rabbit IgG 1:1000 Cell Signaling
JAK1 (120 kDa)	1:500, BSA 5% in TBS-t Cell Signaling ID: AB_2128499	HRP conjugated anti-rabbit IgG 1:500 Cell Signaling
pp38 Thr180/Tyr182 (43 kDa)	1:500, BSA 5% in TBS-t Cell Signaling ID: AB_331641	HRP conjugated anti-rabbit IgG 1:500 Cell Signaling
p38 (43 kDa)	1:1000, BSA 5% in TBS-t Cell Signaling ID: AB_330713	HRP conjugated anti-rabbit IgG 1:2000 Cell Signaling
pERK1/2 Thr202/Tyr204 (42/44 kDa)	1:1000, 3% nonfat dry milk in TBS-t Cell Signaling ID: AB_2315112	HRP conjugated anti-rabbit IgG 1:2000 Cell Signaling
ERK1/2 (42/44 kDa)	1:5000 1% nonfat dry milk in TBS-t Santa Cruz Biotech ID: AB_2140110	HRP conjugated anti-rabbit IgG 1:5000 Cell Signaling
β-Actin (43 kDa)	1:10,000 3% nonfat dry milk in TBS-t Sigma-Aldrich ID: AB_476744	HRP conjugated anti-mouse IgG 1:20,000 Sigma-Aldrich

Abbreviations: IL-6, interleukin-6; STAT, signal transducer and activator of transcription; SOCS, suppressor of cytokine signaling; JAK, Janus kinase; BSA, bovine serum albumin; Ig, immunoglobulin; HRP, horseradish peroxidase.

## Data Availability

The data presented in this study are available on request from the corresponding author.

## Supplementary Materials

The following supporting information can be downloaded at: https://www.mdpi.com/article/10.3390/ijms232012453/s1.
