# Supplementary material for: Involvement of the IL-6 Signaling Pathway in the Anti-Anhedonic Effect of the Antidepressant Agomelatine in the Chronic Mild Stress Model of Depression
Source: Int J Mol Sci. 2022 Oct 18;23(20):12453. doi: 10.3390/ijms232012453 (PMC9604470; doi:10.3390/ijms232012453)
Supplement: Supplementary file 1 [file ijms-23-12453-s001.zip › ijms-1926709-supplementary.pdf]

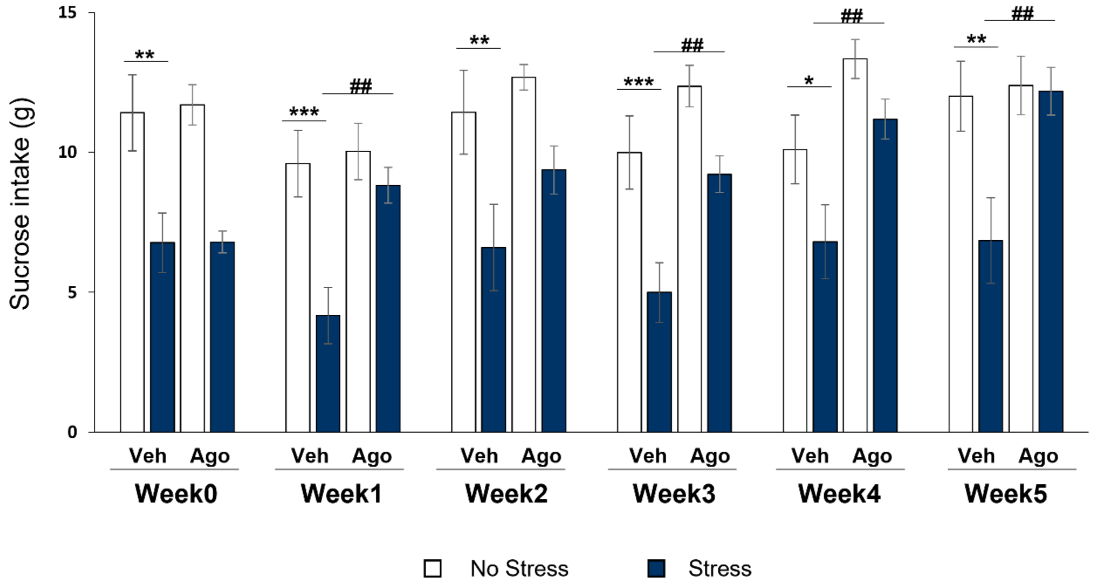


**Supplementary Figure S1** - Effect of Agomelatine treatment on the sucrose intake of animals exposed to 7 weeks of chronic mild stress (CMS). The sucrose intake was measured weekly in rats (n= 10 each experimental group) exposed to CMS and chronically treated Agomelatine for 5 weeks. The data are expressed as grams (g) of sucrose intake, as the mean ± SEM. *P < 0.05; **P < 0.01; ***P < 0.001 vs. No Stress/Vehicle; ##P < 0.01 vs. Stress/Vehicle (two-way ANOVA with PLSD).

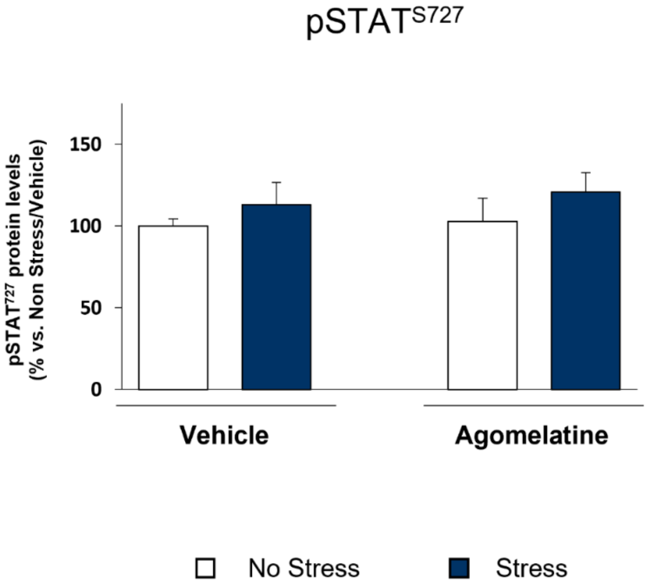


**Supplementary Figure S2** - Effect of CMS exposure and agomelatine treatment on the nuclear phosphorylation of STAT3 (Ser727).

The protein levels of pSTAT3 at serine 727 were assessed in the prefrontal cortex of rats exposed to CMS treatment with agomelatine. The data, expressed as percentage versus unstressed rats treated with vehicle (No Stress/Vehicle, set at 100%) are the mean ± SEM of at least 6 independent determinations and are presented as a ratio between the phosphorylated and the total forms of the proteins examined. Two-way ANOVA revealed no statistically significant effects.

| Target | ANOVA | | Experimental Group | % change (vs. CTRL) | P-Value vs. CTRL | P-Value vs. CMS | Figure |
|---|---|---|---|---|---|---|---|
| *Il-6* | Agomelatine | F(1,32) = 7.892 p= 0.009 | Agomelatine | -5% | - | - | Fig. 2A |
| | Stress | - | CMS | +51% | 0.004** | - | |
| | Agomelatine x Stress | F(1,32) = 9.868 p= 0.004 | CMS+AGO | -16% | - | 0.0006### | |
| IL-6 | Agomelatine | - | Agomelatine | -10% | - | - | Fig. 2C |
| | Stress | - | CMS | +38% | - | - | |
| | Agomelatine x Stress | - | CMS+AGO | -9% | - | - | |
| pJAK1/JAK1 Y1022/1023 | Agomelatine | F(1,32) = 4.094 p= 0.053 | Agomelatine | +11% | - | - | Fig. 2D |
| | Stress | - | CMS | +34% | 0.0002*** | - | |
| | Agomelatine x Stress | F(1,32) = 15.135 p= 0.001 | CMS+AGO | -1% | - | 0.0004### | |
| pSTAT3 Y705 (Cytosol) | Agomelatine | - | Agomelatine | +9 | | | Fig. 3A |
| | Stress | - | CMS | +37 | 0.013* | | |
| | Agomelatine x Stress | F(1,36) = 6.764 p= 0.014 | CMS+AGO | -4 | | 0.005## | |
| STAT3 (Cytosol) | Agomelatine | - | Agomelatine | -3 | - | - | Fig. 3C |
| | Stress | - | CMS | +9 | - | - | |
| | Agomelatine x Stress | - | CMS+AGO | -11 | - | - | |
| pSTAT3 Y705 (Nucleus) | Agomelatine | - | Agomelatine | +83 | 0.00009*** | - | Fig. 3B |
| | Stress | - | CMS | +79 | 0.012** | - | |
| | Agomelatine x Stress | F(1,27) = 23.212 p= 0.00007 | CMS+AGO | +27 | - | 0.043# | |
| STAT3 (Nucleus) | Agomelatine | - | Agomelatine | +4 | - | - | Fig. 3D |
| | Stress | - | CMS | -8 | - | - | |
| | Agomelatine x Stress | - | CMS+AGO | -4 | - | - | |
| *Socs3* | Agomelatine | F(1,33) = 11.077 p= 0.002 | Agomelatine | +49 | 0.006** | - | Fig. 4A |
| | Stress | F(1,33) = 6.216 p= 0.019 | CMS | +30 | 0.035* | - | |
| | Agomelatine x Stress | - | CMS+AGO | +69 | 0.00002*** | - | |
| SOCS3 | Agomelatine | F(1,34) = 10.958 p= 0.002 | Agomelatine | +61 | 0.0005*** | - | Fig. 4B |
| | Stress | - | CMS | +35 | 0.032* | - | |

| | | | | | | | |
|---|---|---|---|---|---|---|---|
| | Agomelatine x Stress | F(1,34) = 4,681<br>p= 0.039 | CMS+AGO | +48 | 0.004** | - | |
| pp38/p38<br>T180/Y182 | Agomelatine | F(1,32) = 3.983<br>p= 0.056 | Agomelatine | +46 | 0.008** | - | Fig. 5A |
| | Stress | - | CMS | +39 | 0.032* | - | |
| | Agomelatine x Stress | F(1,32) = 3.707<br>p= 0.064 | CMS+AGO | +39 | 0.024* | - | |
| pERK1/ERK1<br>T202/Y204 | Agomelatine | - | Agomelatine | +10 | - | - | Fig. 5B |
| | Stress | - | CMS | +58 | 0.050* | - | |
| | Agomelatine x Stress | F(1,30) = 8.406<br>p= 0.008 | CMS+AGO | -33 | - | 0.004## | |
| pERK2/ERK2<br>T185/Y187 | Agomelatine | - | Agomelatine | +48 | 0.003** | - | Fig. 5C |
| | Stress | F(1,30) = 6.730<br>p= 0.015 | CMS | +77 | 0.00005*** | - | |
| | Agomelatine x Stress | F(1,30) = 17.778<br>p=0.0003 | CMS+AGO | +30 | - | 0.010## | |
| *Bcl-xl* | Agomelatine | F(1,37) = 16.929<br>p= 0.0002 | Agomelatine | +17% | 0.044* | - | Fig. 5D |
| | Stress | - | CMS | -11% | - | - | |
| | Agomelatine x Stress | - | CMS+AGO | +35% | - | 0.0009### | |

**Supplementary Table S1**: The results of the molecular analyses are presented in terms of main effects of the ANOVA analysis, percentual changes between groups and respective p values. Figure S1: Effect of CMS exposure and agomelatine treatment on the nuclear phosphorylation of STAT3 (Ser727). *P < 0.05; **P < 0.01; ***P < 0.001 vs. No Stress/Vehicle; #P < 0.05; ##P < 0.01; ###P < 0.001 vs. Stress/Vehicle (two-way ANOVA with PLSD).

Figure S3

Western Blot analysis of **IL-6** in the prefrontal cortex of rats exposed to 7weeks of CMS treated or not with the antidepressant agomelatine

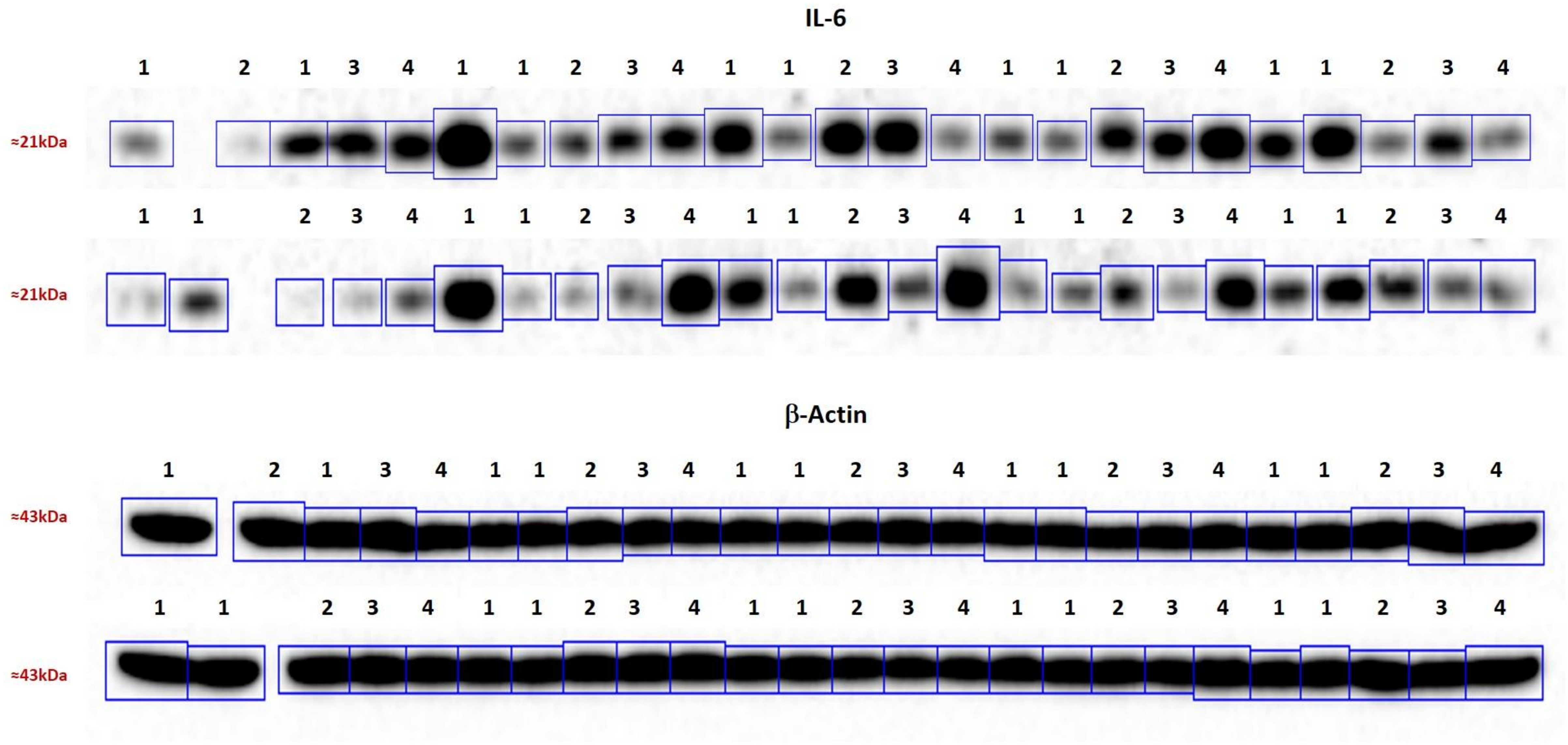


*Legend: 1= No Stress/Vehicle; 2= Stress/Vehicle; 3= No Stress/Agomelatine; 4= Stress/Agomelatine.*

Figure S4

# Western Blot analysis of pJAK1$^{Y1022/1023}$ in the prefrontal cortex of rats exposed to 7weeks of CMS treated or not with the antidepressant agomelatine

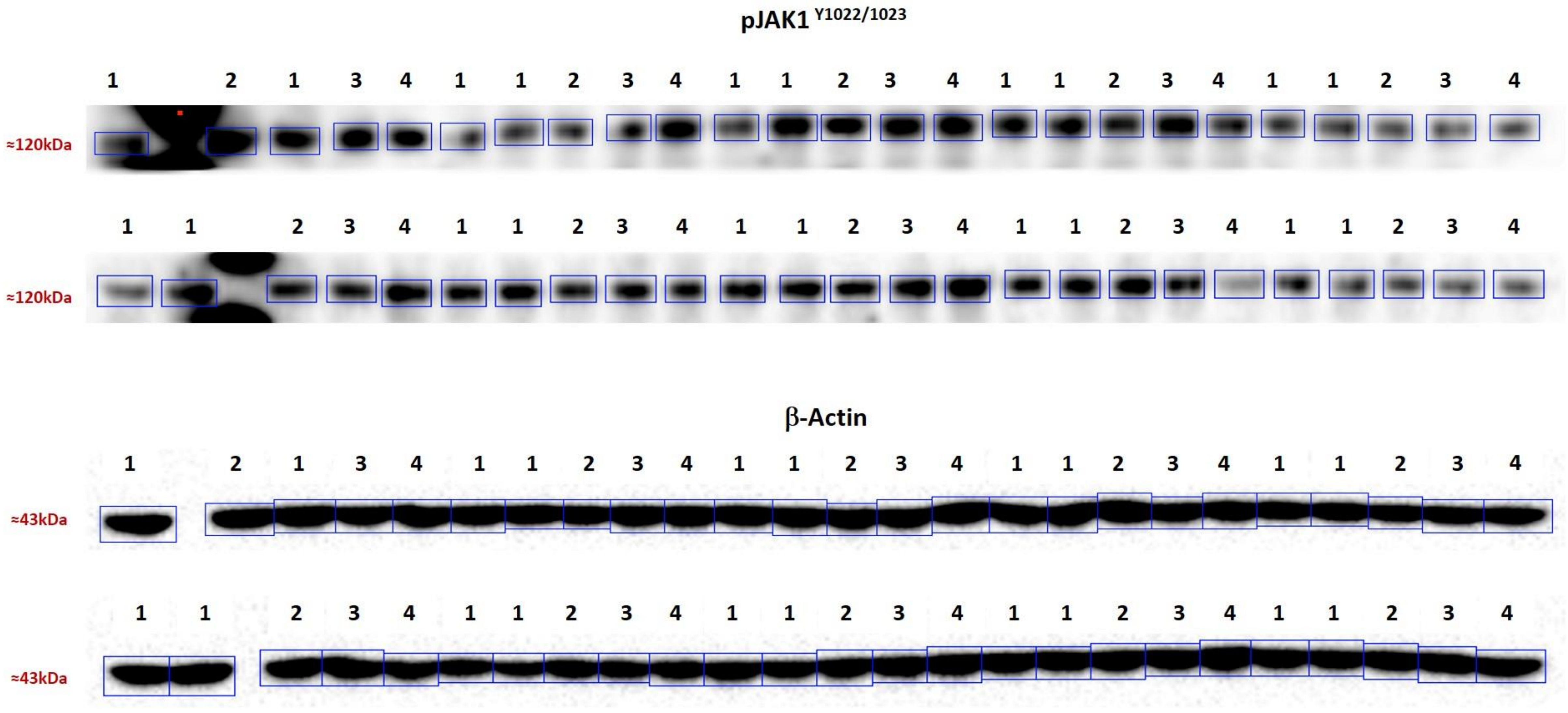


*Legend: 1= No Stress/Vehicle; 2= Stress/Vehicle; 3=No Stress/Agomelatine; 4=Stress/Agomelatine.*

Figure S5

Western Blot analysis of **JAK1** in the prefrontal cortex of rats exposed to 7weeks of CMS treated or not with the antidepressant agomelatine

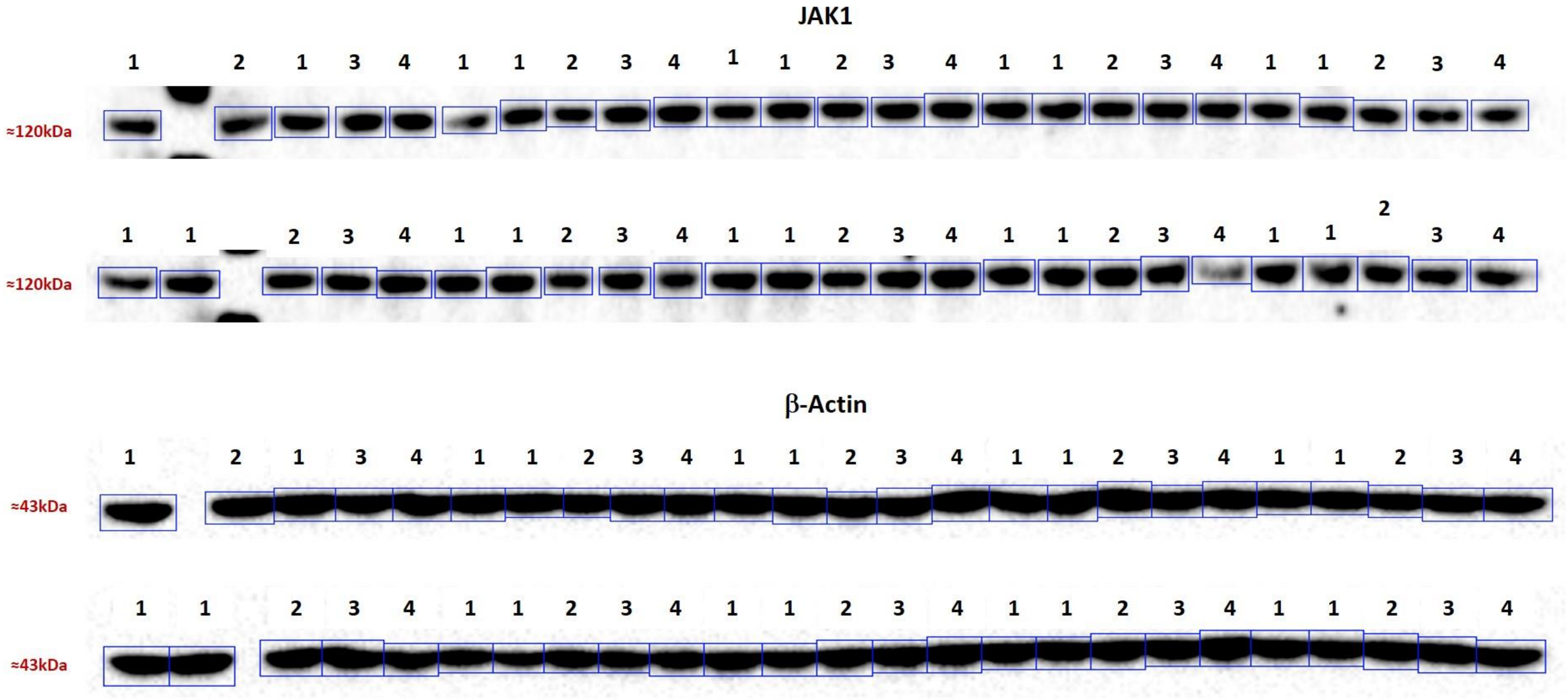


*Legend: 1= No Stress/Vehicle; 2= Stress/Vehicle; 3=No Stress/Agomelatine; 4=Stress/Agomelatine.*

Figure S6

Western Blot analysis of **pSTAT3$^{Y705}$** in the prefrontal cortex of rats exposed to 7weeks of CMS treated or not with the antidepressant agomelatine

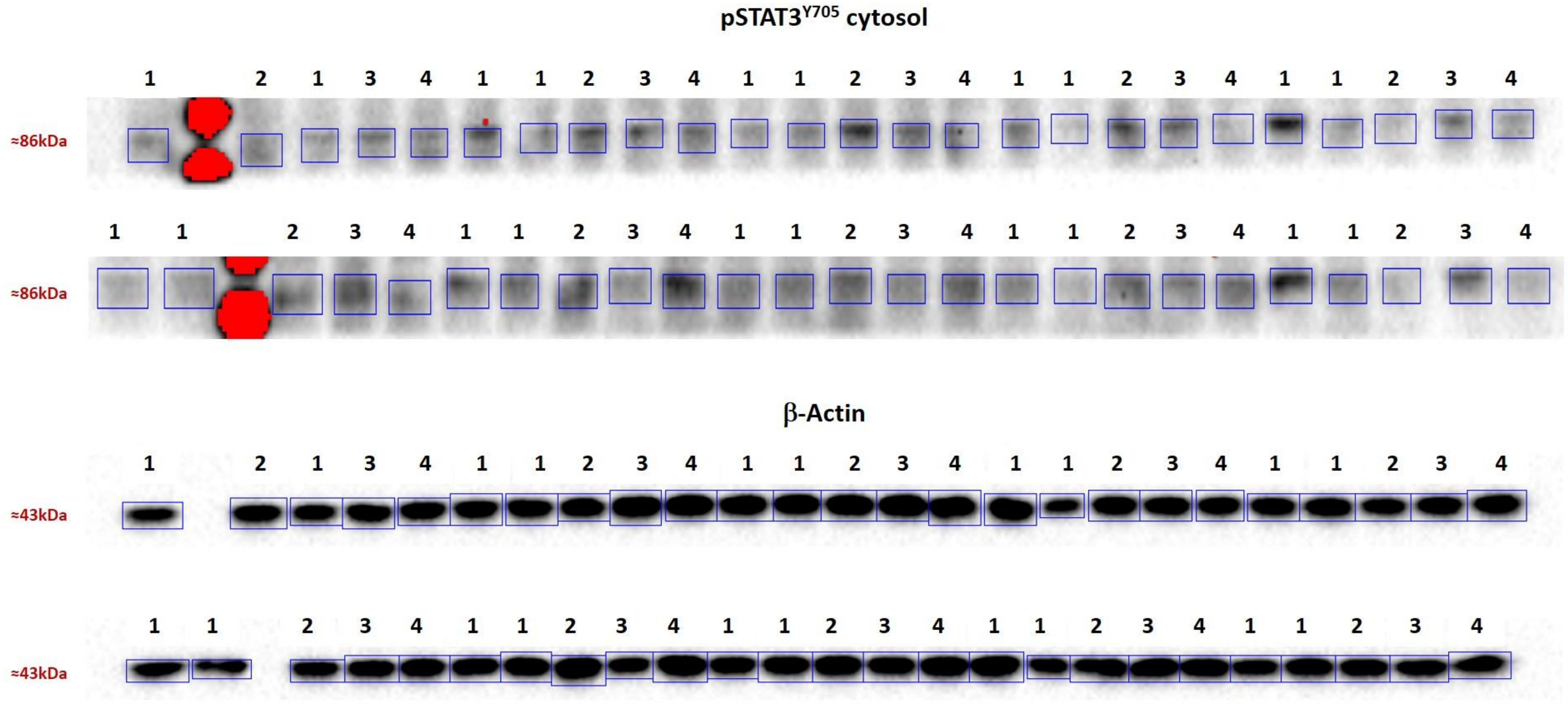


***Legend: 1= No Stress/Vehicle; 2= Stress/Vehicle; 3= No Stress/Agomelatine; 4= Stress/Agomelatine.***

Figure S7

Western Blot analysis of **STAT3** in the prefrontal cortex of rats exposed to 7weeks of CMS treated or not with the antidepressant agomelatine

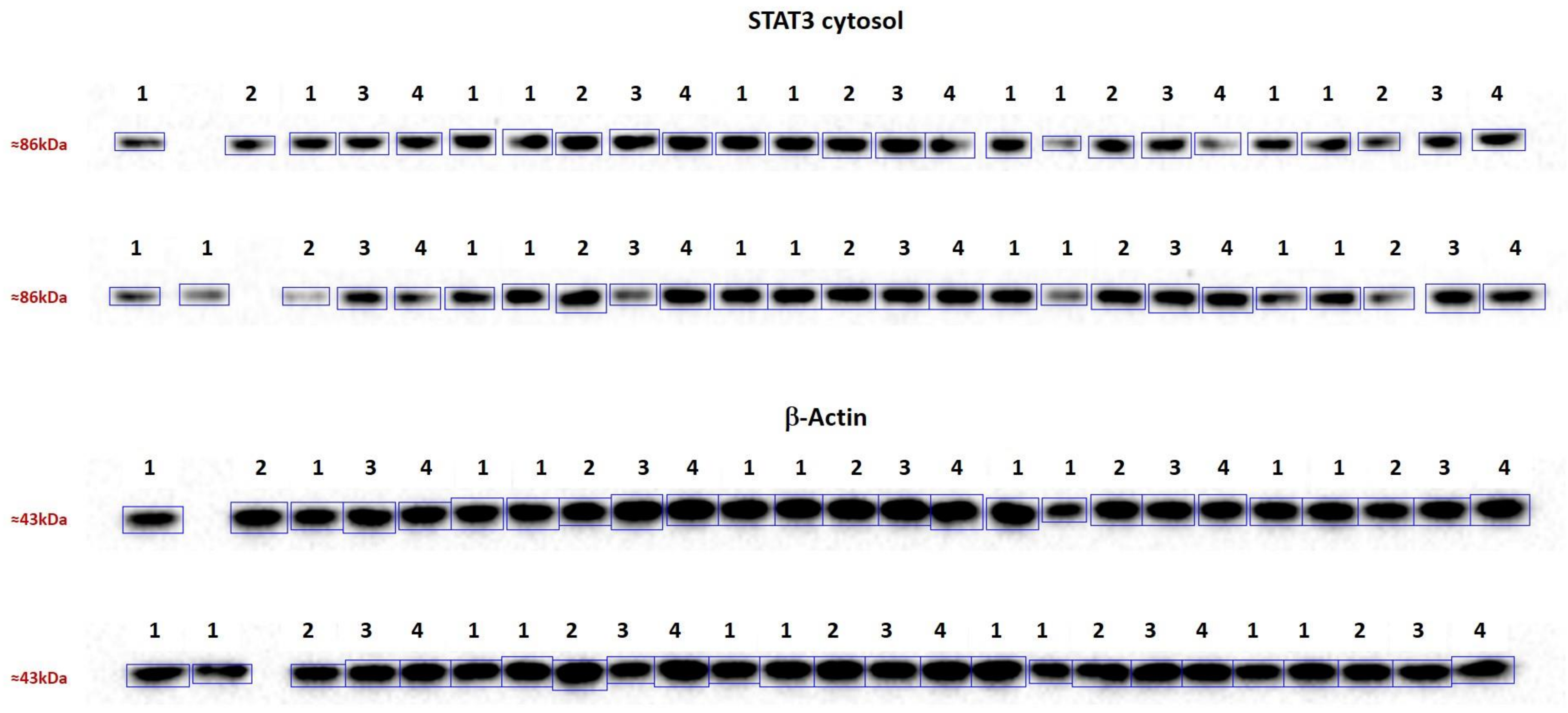


*Legend: 1= No Stress/Vehicle; 2= Stress/Vehicle; 3= No Stress/Agomelatine; 4= Stress/Agomelatine.*

Figure S8

Western Blot analysis of **pSTAT3$^{Y705}$** in the prefrontal cortex of rats exposed to 7weeks of CMS treated or not with the antidepressant agomelatine

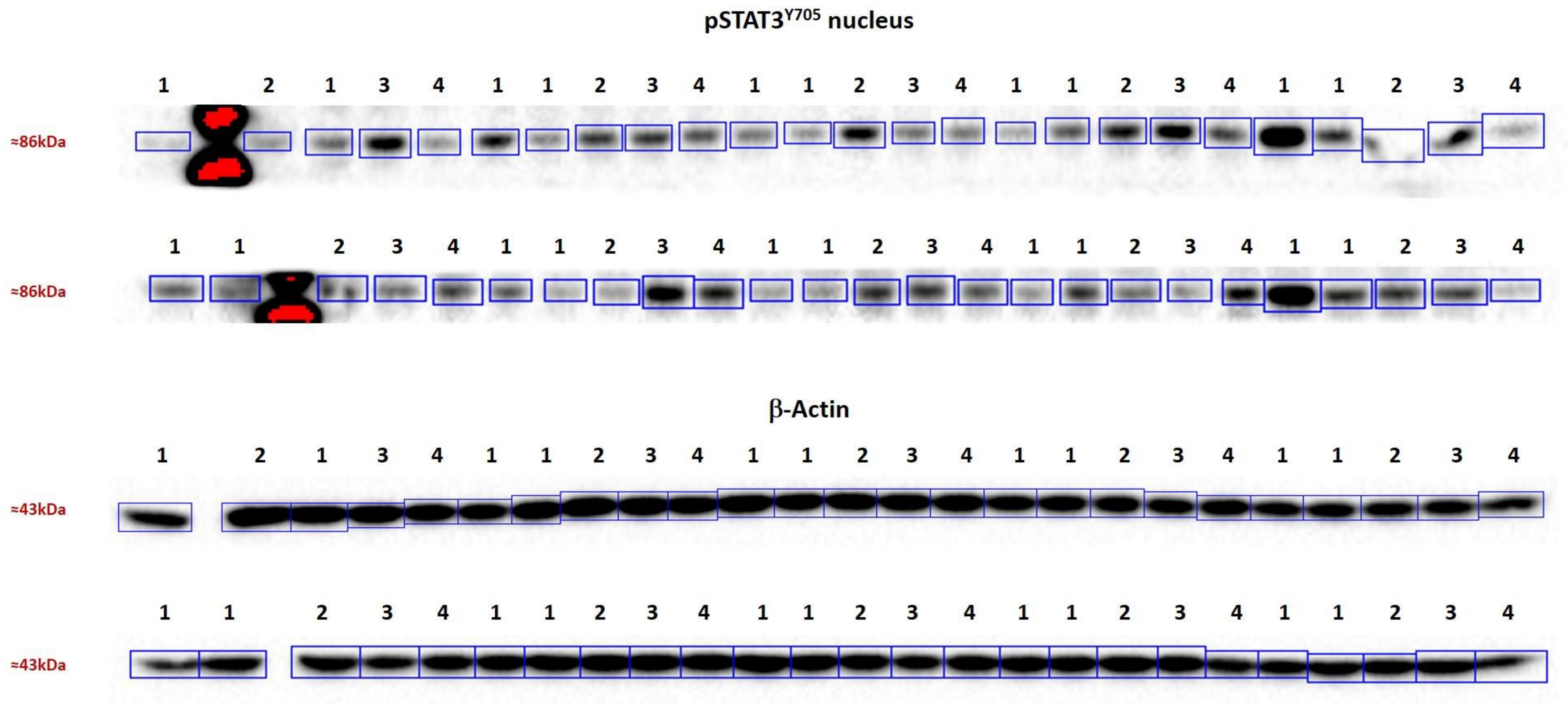


*Legend: 1= No Stress/Vehicle; 2= Stress/Vehicle; 3= No Stress/Agomelatine; 4= Stress/Agomelatine.*

Figure S9

Western Blot analysis of **STAT3** in the prefrontal cortex of rats exposed to 7weeks of CMS treated or not with the antidepressant agomelatine

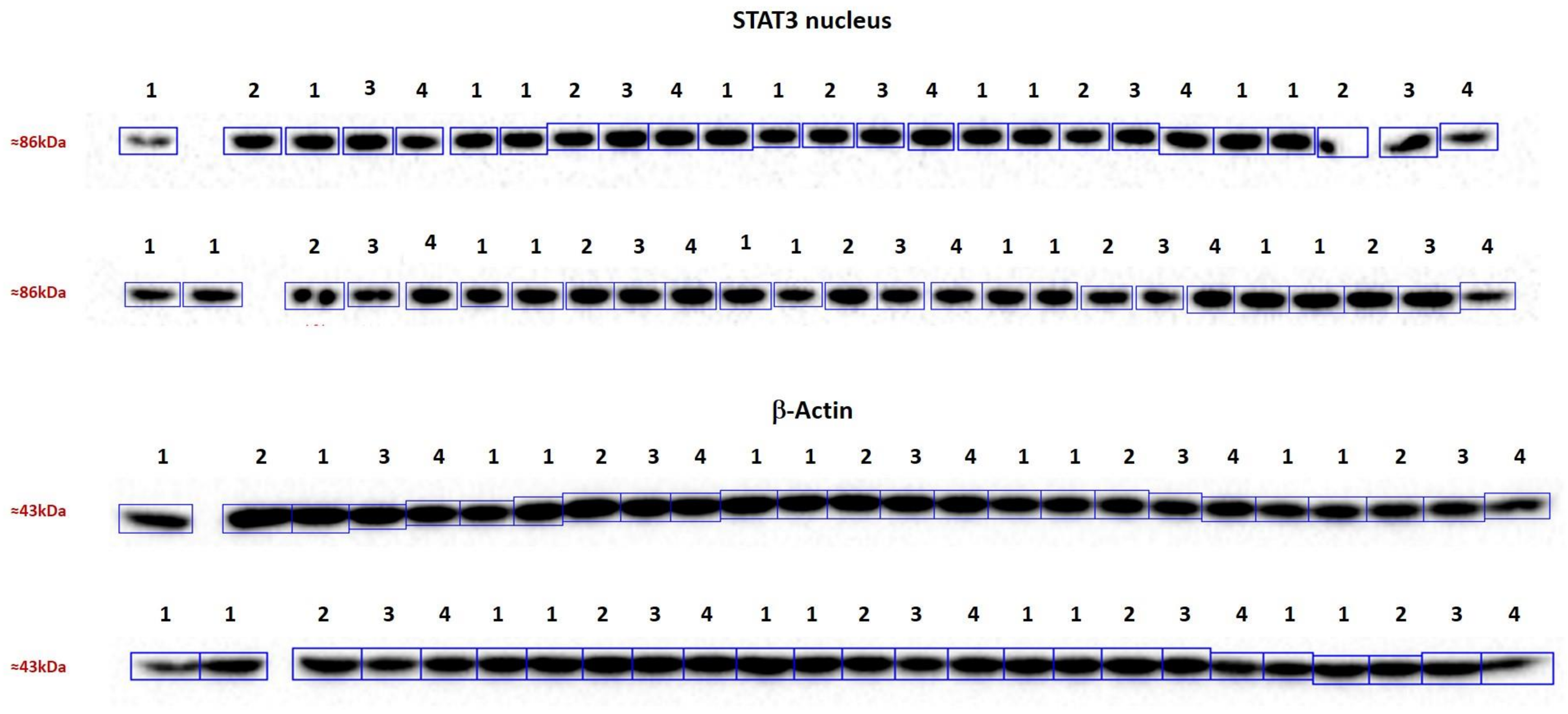


*Legend: 1= No Stress/Vehicle; 2= Stress/Vehicle; 3= No Stress/Agomelatine; 4= Stress/Agomelatine.*

Figure S10

Western Blot analysis of **SOCS3** in the prefrontal cortex of rats exposed to 7weeks of CMS treated or not with the antidepressant agomelatine

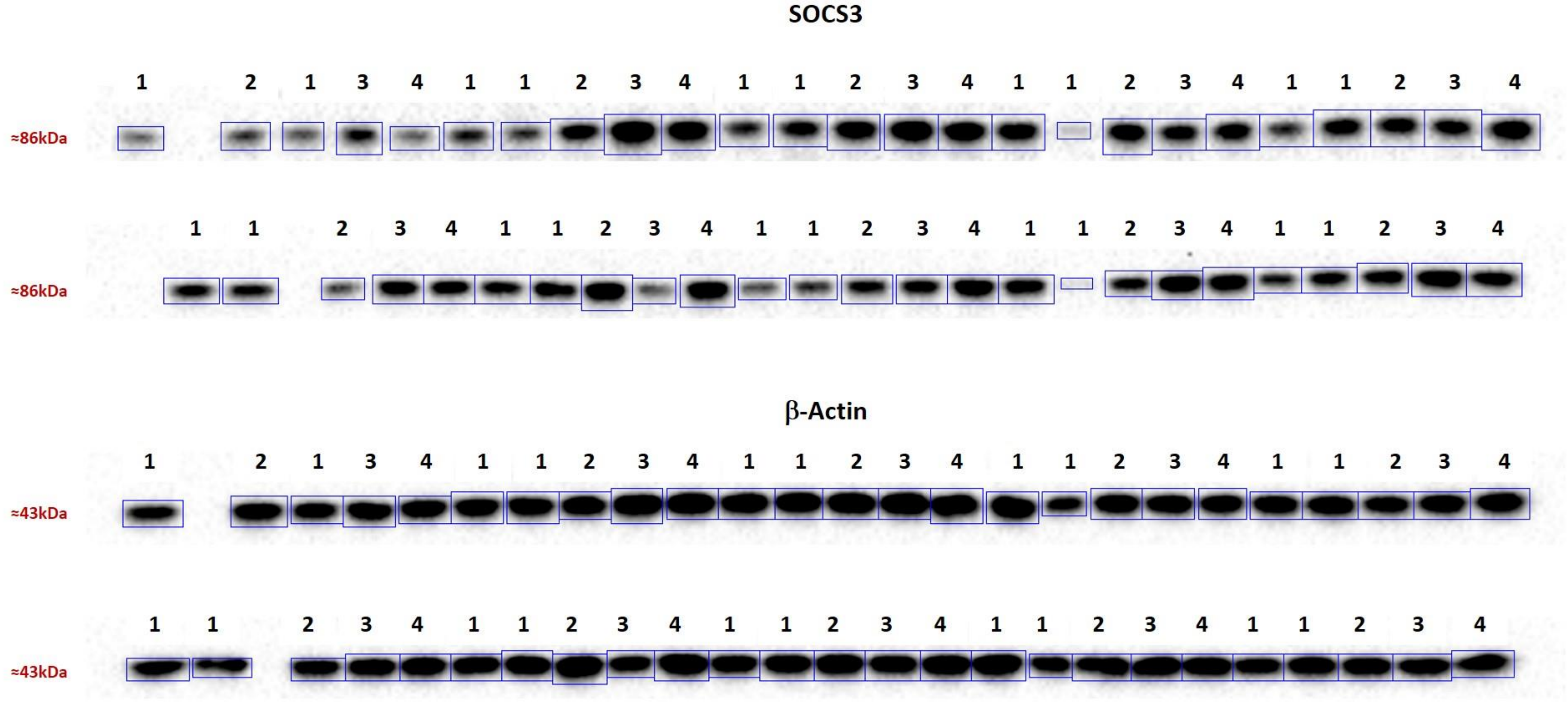


***Legend: 1= No Stress/Vehicle; 2= Stress/Vehicle; 3= No Stress/Agomelatine; 4= Stress/Agomelatine..***

Figure S11

Western Blot analysis of **pp38**$^{T180/Y182}$ in the prefrontal cortex of rats exposed to 7weeks of CMS treated or not with the antidepressant agomelatine

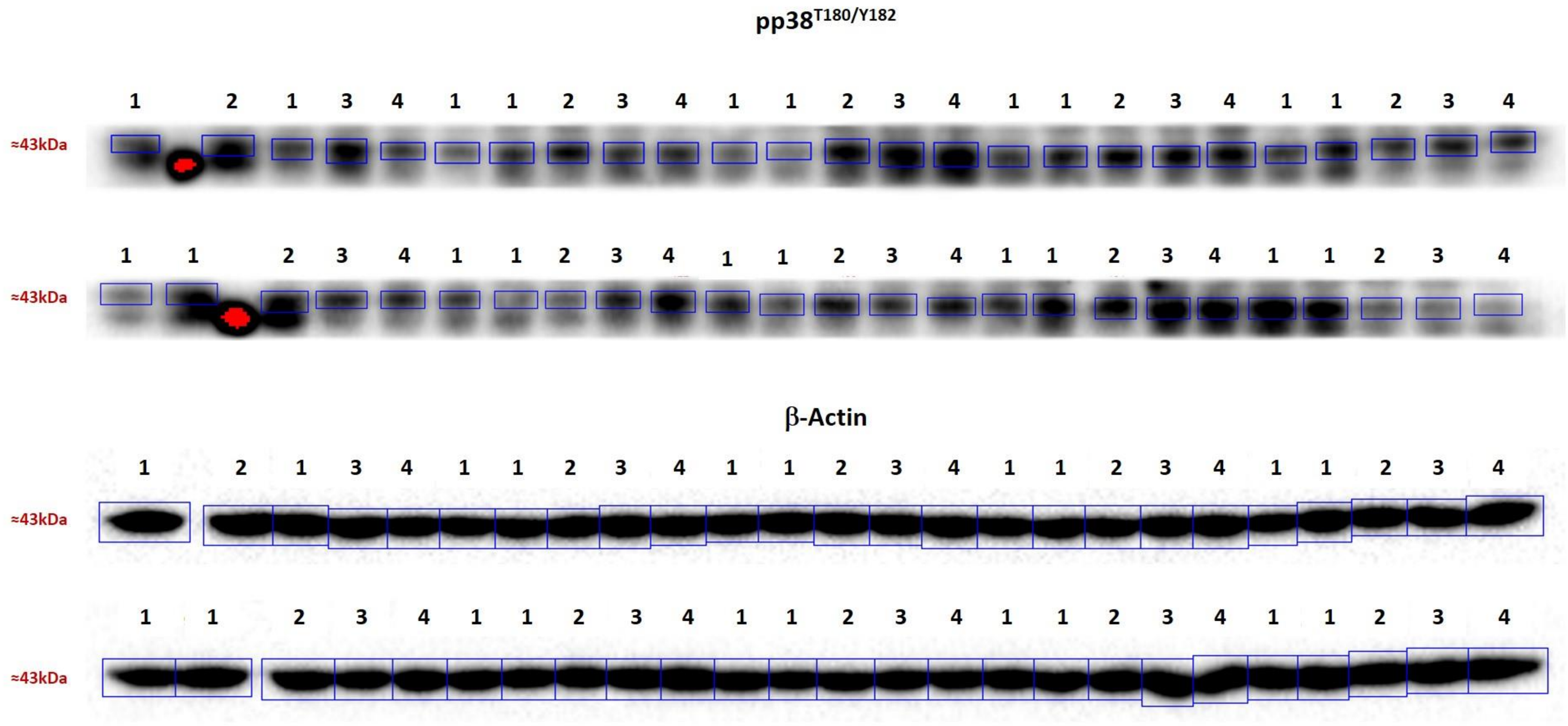


*Legend: 1= No Stress/Vehicle; 2= Stress/Vehicle; 3= No Stress/Agomelatine; 4= Stress/Agomelatine.*

Figure S12

Western Blot analysis of **p38** in the prefrontal cortex of rats exposed to 7weeks of CMS treated or not with the antidepressant agomelatine

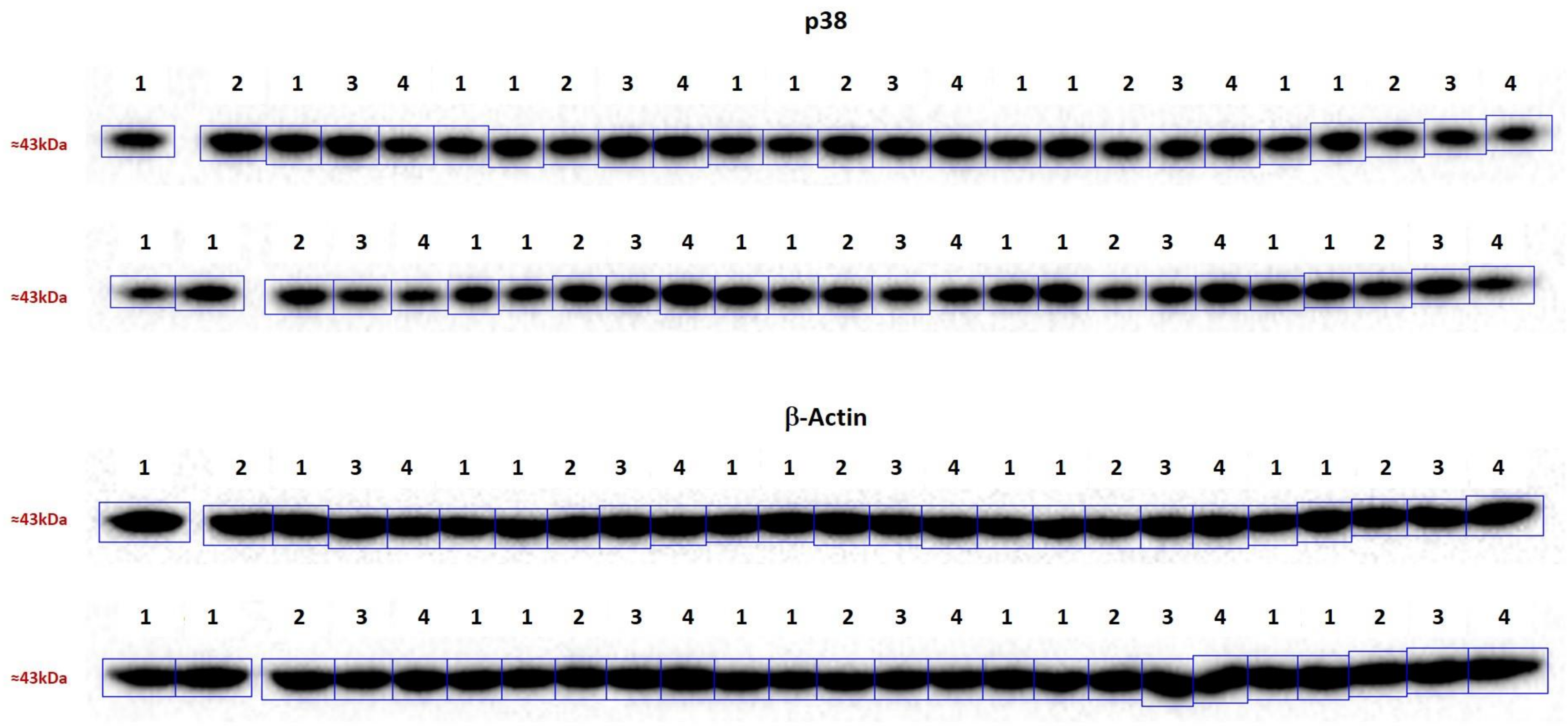


***Legend: 1= No Stress/Vehicle; 2= Stress/Vehicle; 3= No Stress/Agomelatine; 4= Stress/Agomelatine.***

Figure S13

Western Blot analysis of **pERK1**$^{T202/Y204}$ in the prefrontal cortex of rats exposed to 7weeks of CMS treated or not with the antidepressant agomelatine

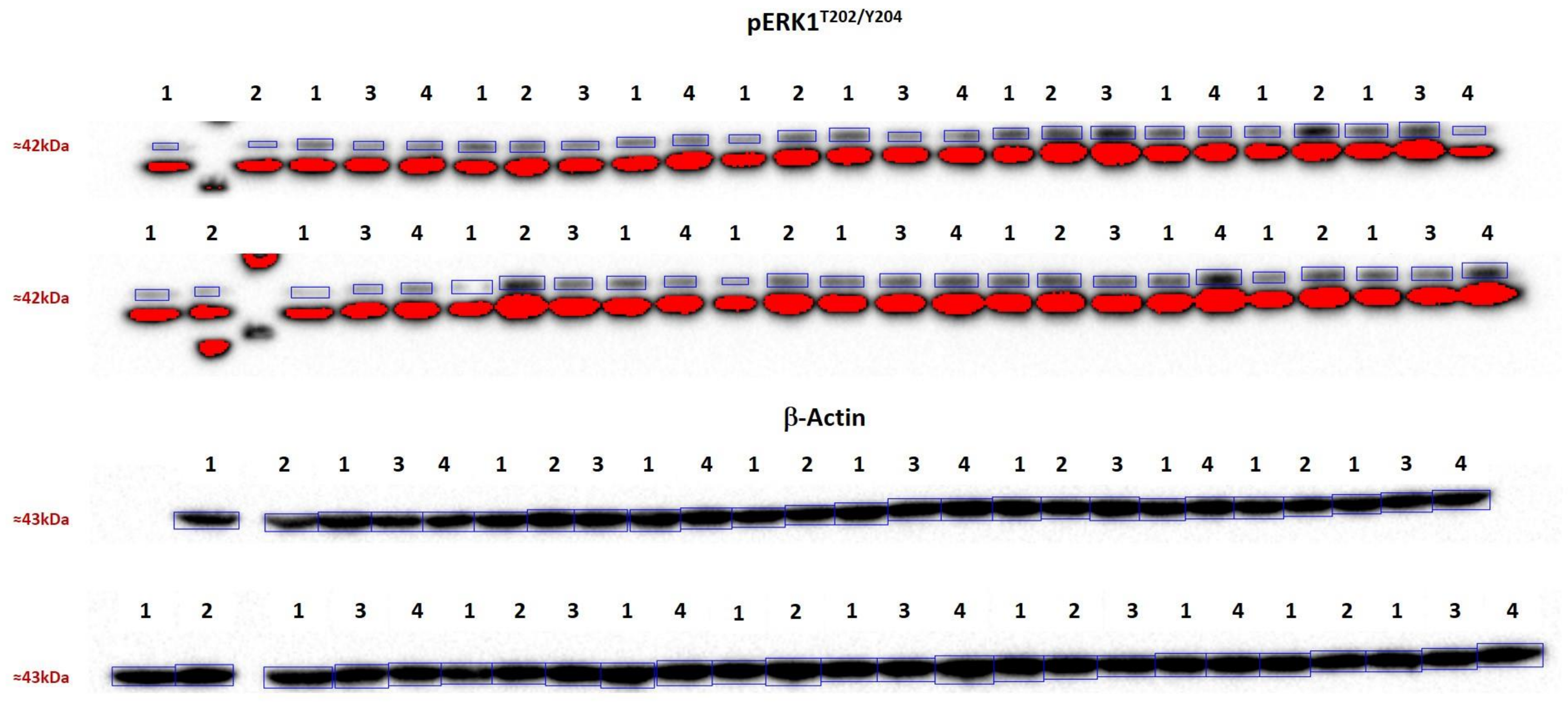


*Legend: 1= No Stress/Vehicle; 2= Stress/Vehicle; 3= No Stress/Agomelatine; 4= Stress/Agomelatine.*

Figure S14

# Western Blot analysis of **ERK1** in the prefrontal cortex of rats exposed to 7weeks of CMS treated or not with the antidepressant agomelatine

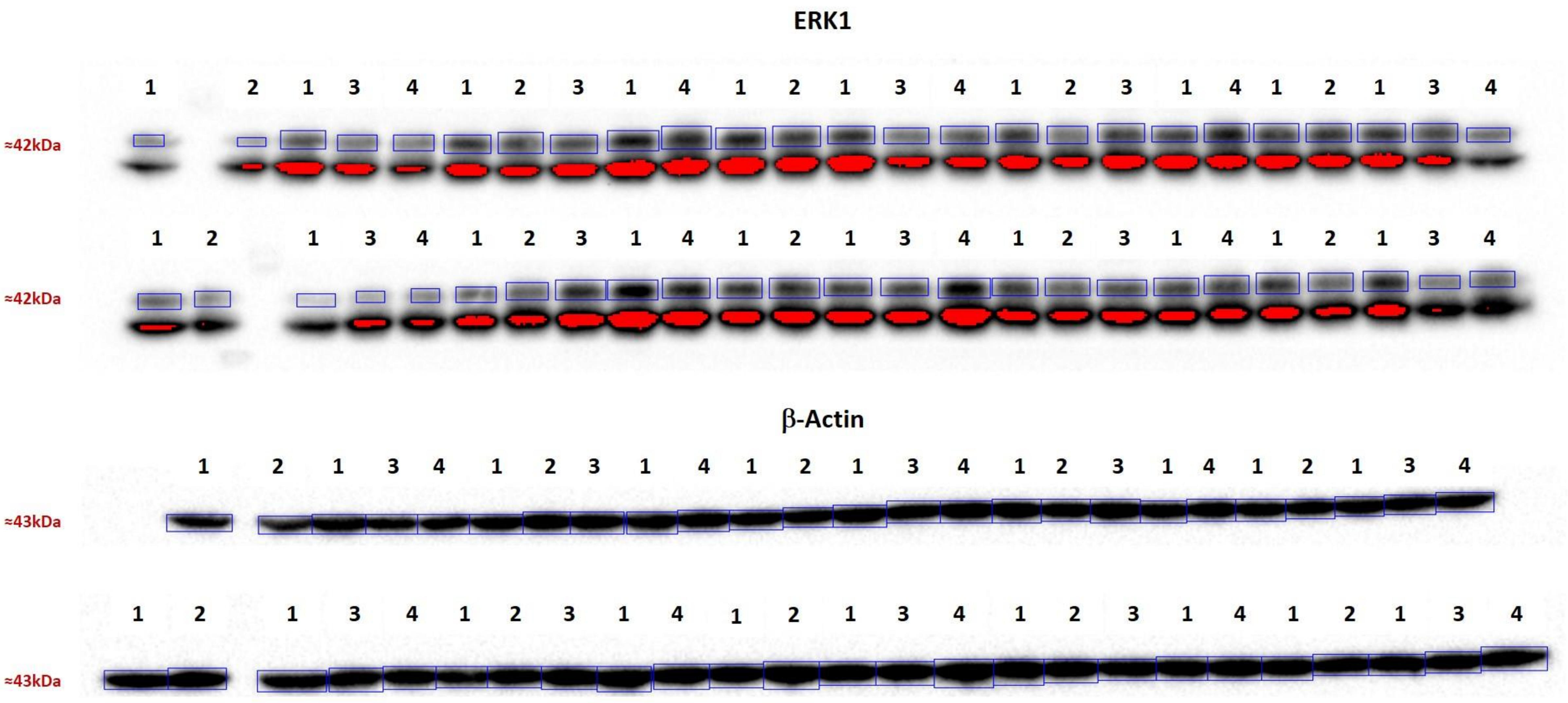


*Legend: 1= No Stress/Vehicle; 2= Stress/Vehicle; 3= No Stress/Agomelatine; 4= Stress/Agomelatine.*

Figure S15

# Western Blot analysis of **pERK2$^{T202/Y204}$** in the prefrontal cortex of rats exposed to 7weeks of CMS treated or not with the antidepressant agomelatine

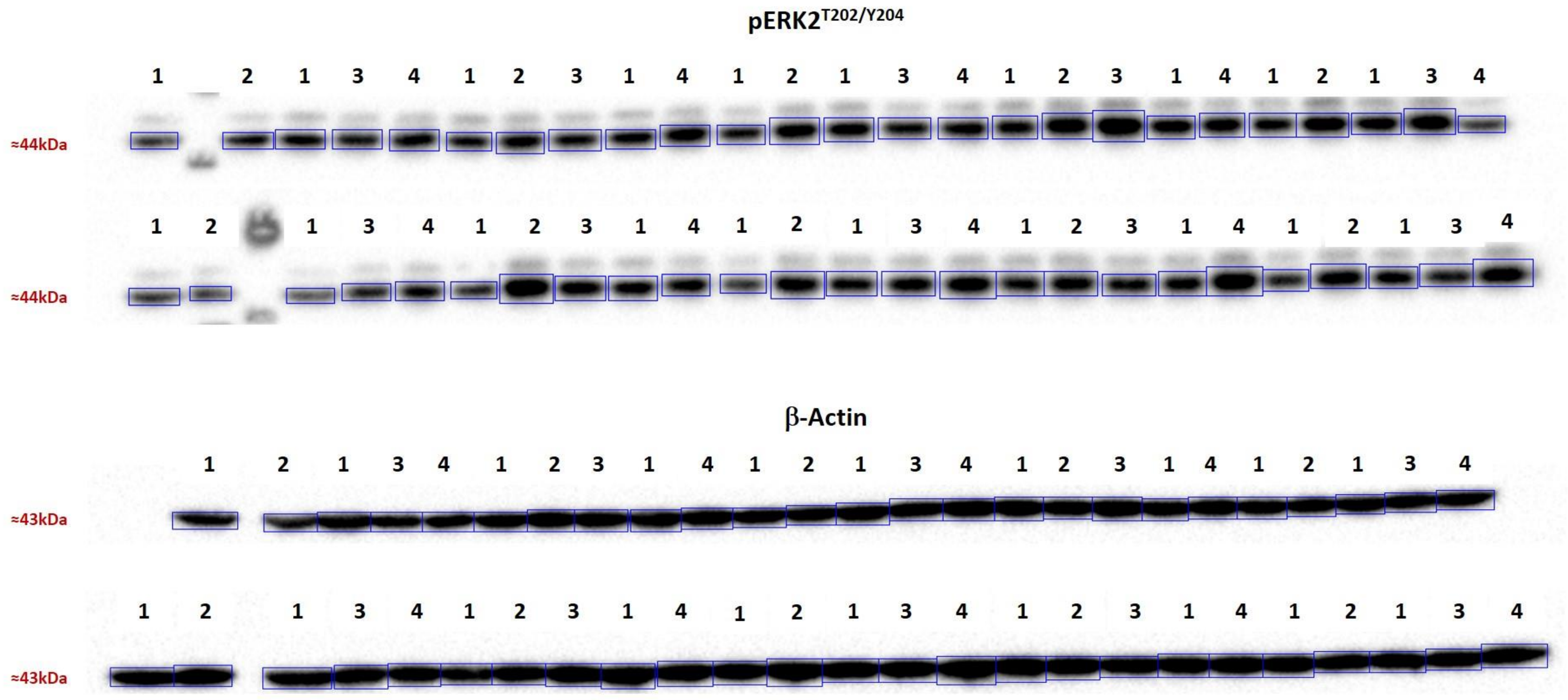


*Legend: 1= No Stress/Vehicle; 2= Stress/Vehicle; 3= No Stress/Agomelatine; 4= Stress/Agomelatine.*

Figure S16

Western Blot analysis of **ERK2** in the prefrontal cortex of rats exposed to 7weeks of CMS treated or not with the antidepressant agomelatine

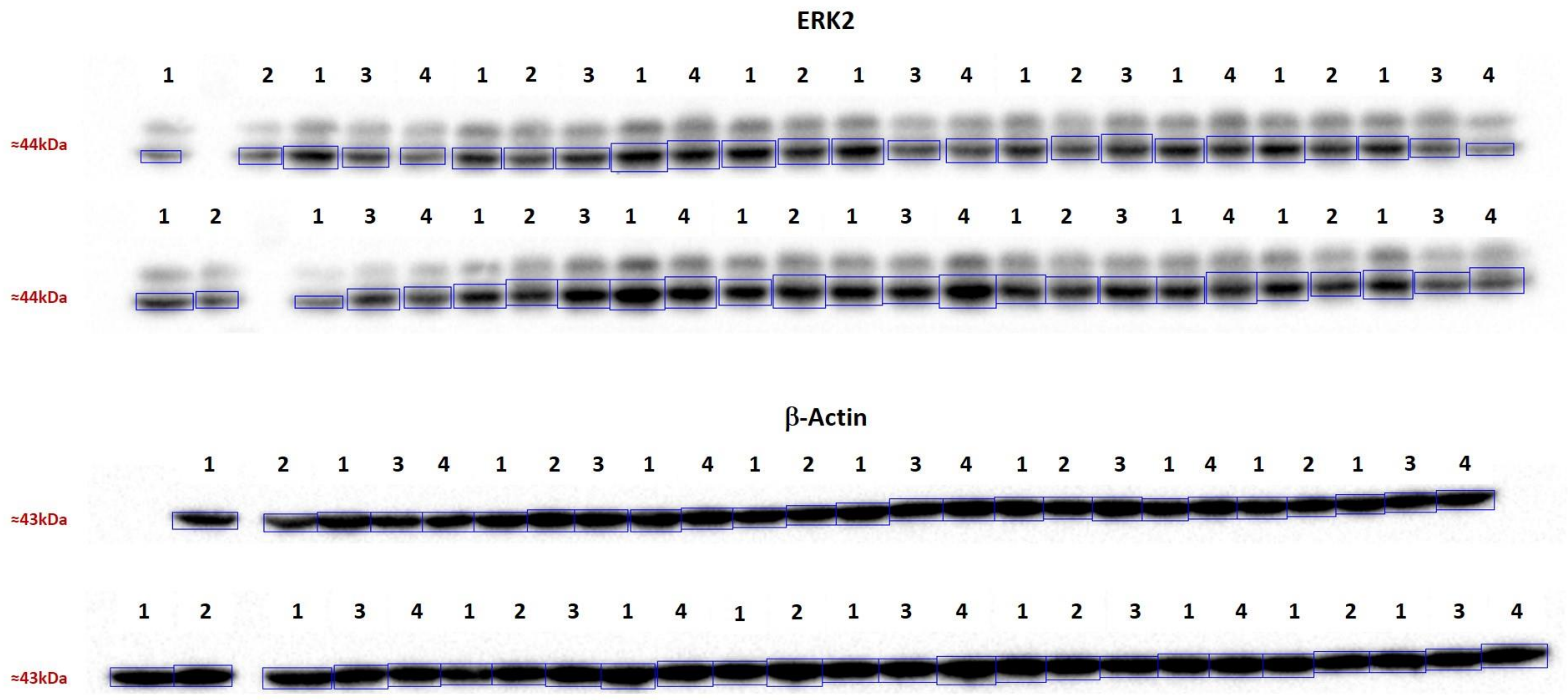


***Legend: 1= No Stress/Vehicle; 2= Stress/Vehicle; 3= No Stress/Agomelatine; 4= Stress/Agomelatine.***

Figure S17

# Western Blot analysis of **pSTAT3**[S727] in the prefrontal cortex of rats exposed to 7weeks of CMS treated or not with the antidepressant agomelatine

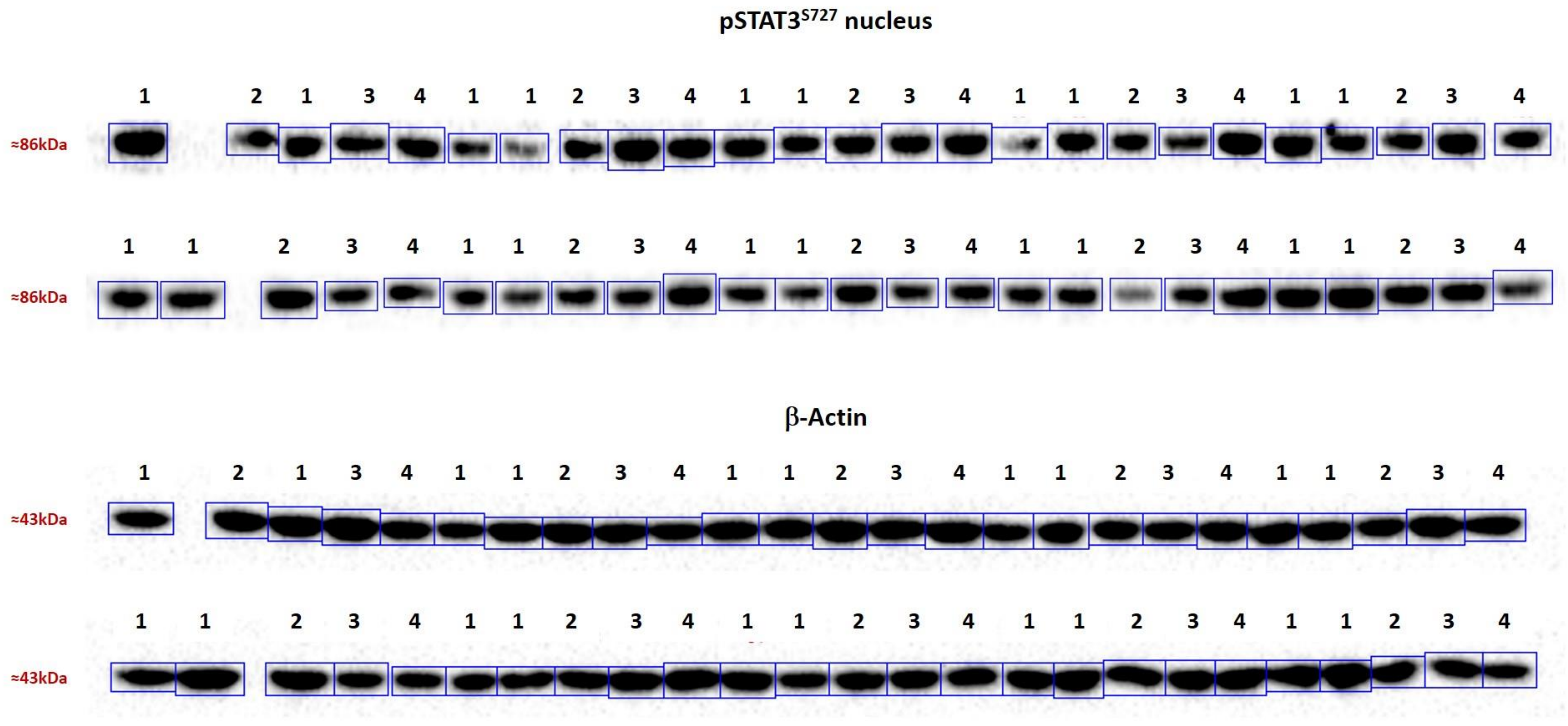


*Legend: 1= No Stress/Vehicle; 2= Stress/Vehicle; 3= No Stress/Agomelatine; 4= Stress/Agomelatine.*
